# Reinforcement learning-driven optimal capital allocation for intelligent finance

**DOI:** 10.1371/journal.pone.0348010

**Published:** 2026-10-20

**Authors:** Yan Luo, Mingchun Qi, Mingshan Cheng

**Affiliations:** School of Intelligent Logistics and Supply Chain, Sichuan Vocational and Technical College, Suining, Sichuan, China; Beijing Technology and Business University, CHINA

## Abstract

Optimal capital allocation in financial markets has long been a challenging task due to the dynamic nature of market conditions, risk factors, and the complexity of financial data. Traditional methods of portfolio management often fail to adapt to market shifts and do not fully leverage potential of modern machine learning techniques. To develop a model that dynamically optimizes capital allocation, leveraging reinforcement learning (RL), with focus on risk management, stability, and adaptability. Proposed model, Dynamic Actor-Critic (DAC)-enriched Clipped Proximal Policy Optimization (DAC-CPPO) model for portfolio optimization. A Dynamic Actor-Critic (DAC)-enriched Clipped Proximal Policy Optimization (DAC-CPPO) model is developed for portfolio optimization. The Dynamic Actor-Critic module learns optimal portfolio policies by evaluating actions and maximizing long-term returns, while the CPPO mechanism ensures stable policy updates and risk-controlled portfolio optimization. The Intelligent Finance Assets dataset contains 7,000 records with 32 features, whereas the Massive Yahoo Finance dataset includes 603k records with 9 features, collected from publicly available Kaggle sources for financial analysis. Normalization and data cleaning techniques for smooth data integration and model readiness, and LDA extracts discriminative financial features to improve portfolio allocation prediction performance. Experimental settings are implemented using Python. Proposed DAC-CPPO model, achieving 97.6% accuracy, 0.074 MAE, 0.081 RMSE, 1.91 Sharpe ratio, 1.12 Cumulative return, and 0.14 Volatility, demonstrates superior risk-adjusted performance, offering higher returns while managing market risks effectively compared to other existing models. The DAC-CPPO model offers a robust solution for practical capital allocation by combining advanced RL techniques, ensuring effective decision-making while managing risks in dynamic financial environments.

## 1. Introduction

The optimal capital allocation in the financial markets is motivated by the efficient allocation of available investment capital among various assets to maximize the expected return for a given level of risk for an investor. The scientific basis of this problem lies in the Modern Portfolio Theory introduced by Harry Markowitz, which proved formally that there is a trade-off between risk and return that can be represented by the variance as a common risk indicator [1,2]. Different macroeconomic variables influence the behavior of financial markets as complex adaptive systems. Globally, these are shaped by geopolitical events, feedbacks resulting from behavioral biases, international correlations and liquidity [3]. Prices of financial assets are governed by a stochastic behavior, following volatility clustering, power law tail distributions and long-range volatility dependency, complicating forecasting. Moreover, financial data are non-stationary, presenting unstable statistical properties over time [4,5]. The correlations among assets are dynamic and tend to increase in times of uncertainty and systemic risk. The dynamic optimization in asset allocation should be based on a multi-country basis dynamic decision process able to adapt to the continuously changing market structures, risk factors and system-wide interactions among assets [6,7]. Classical portfolio management methods, particularly the mean-variance optimization, are based on assumptions that are well established in the classical statistical analysis, such as normally distributed assets returns and constant asset correlations. In fact, modern financial markets are much more complex than the relatively static world assumed in these models [8]. They are subject to huge economic and geopolitical variations, and they are also exposed to global contextual impacts, passing through several shocks [9,10]. The dark side of this weak relation of the models with economic variation is that they are very dependent on using historic data, facing the problem that the pattern of the historical data may not remain the same in the future. In time of crisis, high volatility and high covariance are expected, incorporating huge distortion in expectations of returns and covariances [11,12]. Traditional models do not take into account transaction costs, market liquidity and behavioral patterns in markets, resulting in being not very dynamic in response to sudden shocks; consequently, they generate suboptimal allocations, where portfolios are more exposed to systemic risks [13,14].

The growing complexity and volatility of modern financial markets make optimal capital allocation increasingly challenging. Traditional approaches and conventional DRL methods often struggle with non-stationary environments, unstable policy updates, high variance, and poor adaptability to sudden market shifts. To address these limitations, the proposed DAC-CPPO reinforcement learning framework integrates asynchronous multi-agent learning with clipped policy optimization. It ensures stable learning, reduced risk exposure, improved convergence, and enhanced risk-adjusted returns for dynamic portfolio management.

The related works review existing methods, analyze their limitations in capital allocation, market adaptability, and risk control, and highlight how the proposed DAC-CPPO model achieves superior stability and performance.

The capital allocation of asset management firms was studied [15] to ensure efficiency and sustainability in the long run. A successful model combining a literature review, case studies, and facts gathered by the world managers was utilized. The findings showed enhanced risk-adjusted returns, transparency of the governance, and strategic alignment due to dynamic budgeting and integration of analytics. Nonetheless, the results were constrained by reliance on secondary data and differences in the market environment. The studied work [16] examined the impact that machine learning, as well as predictive and descriptive analytics, had on capital allocation performance. The outcomes showed that ROI (19.2), market share (4.8), and financial growth were significantly improved (12.6). Limitations to generalizability, however, were imposed by the fact that the data relied on surveys and were limited in geographic scope.

The application of big data technology has a strong impact on the funding system and distribution of funds within the energy business, with the rapid evolution of big data technologies [17]. A big data platform was prepared in an integrated way to optimize financing strategies, provide the positioning of investment, and help discover possible risks. The strategy increased the efficiency of financial management and structural adjustment. Nonetheless, it largely relied on the data quality and technology facilities for implementation. To optimize household financial asset allocation and enhance risk management, an investigation was designed [18] to incorporate real-time data that is generated by the IoT with an optimized K-means++ clustering algorithm. The performance index examined demonstrated better silhouette (0.62) and score (ARI 0.75), and the perspective of diversification and regional risk. Depending on local and behavioral data, however, hindered generalization. Capital allocation was investigated [19] to improve sustainable financial performance in the context of uncertainty. Financial ratios, DCF model, sensitivity analysis, and multi-criteria were united into a conceptual decision model as part of dynamic analytics. Findings showed better transparency and risk-adjusted evaluation. The weaknesses were the complexity of integrating data and the limitations of governance and interpretation of the model. Studies set out to be more efficient and rational in allocating financial resources based on the concepts of big data analytics and machine learning [20]. A hybrid random forest-particle swarm optimization model was used, which enhanced feature selection, parameter optimization, and convergence rate. They found higher accuracy, recall, and F1 scores, but scalability and Practical adaptability were still low. The inconsistency in capital allocation was studied to determine the quality of decisions and the effectiveness of their allocation made with the help of ML-based forecasting and valuation. The correlation, regression, and mediation tests were used to examine the reliability, correlation, regression, and mediation of a quantitative cross-sectional analysis of 162 participants of enterprises [21]. The effects of partial mediation were also very high, so forecast and valuation greatly improved the quality and efficiency of the decisions. Self-reported measures and a cross-sectional design were a limitation to the findings. To enhance supply chain financing using blockchain and large language models [22]. It applies NLP-based LLM integration within blockchain-driven financing platforms. Results show improved information processing and financing efficiency. Limitations include a lack of real-world large-scale validation and dependency on model-driven assumptions, restricting practical deployment and generalization.

Research conducted [23] on the changes in the risk management plans within the investment portfolios during turbulent market states. It was a combination of technology, behavioral finance, and quantitative theories, including algorithmic trading and dynamic allocation of assets. Findings showed better adaptability and minimization of risks than the traditional models. Nevertheless, the sensitivity of the models to severe market shocks and reliance on data remained the main limitations. An AI-aided mechanism could enhance the performance of the portfolio rebalancing shown in [24]. The mechanism collects the financial information, predicts the fluctuation of market using machine learning approach, and dynamically rebalance portfolio. The result could deliver a 12.5% ROI, 1.2 of Sharpe ratio, and 5.2% of drawdown. The performance was, however, sensitive to the stability of data in the market and data quality. In [25], the approach of using AI-based analytics to manage wealth was examined. The strategy utilized machine learning, prediction analytics, and data analysis methods to streamline portfolio management and risk measurement. The outcomes showed enhanced predictive performance, scale performance, and customized asset allocation. However, its constraints were the privacy of data, its dependence on the model, and the danger of over-reliance on automated systems. The use of machine learning to enhance the allocation of cloud resources was also used in [26]. The prediction of resource requirements was based on an LSTM-based model, and its results were compared with Gradient Boosting and Logistic Regression. The performance made 97% accuracy in prediction and enhanced classification performance. Nonetheless, the method relied on quality training data and was not scaled to very dynamic settings. In [27], an algorithm to predict the value of stocks and increase the accuracy of the trading was offered. It combined ANN, LSTM, and DQN models with historical gold stock data and social media to make predictions of the open prices tomorrow. These findings demonstrated better predictive performance as compared to benchmark models. But the drawbacks were reliance on data quality, market volatility, and removal of low complexity.

The performance of AI-based investment models versus traditional methodology of portfolios strategies, like the Modern Portfolio Theory, were investigated [28]. utilizing the historical data and simulation modeling to compare the return, risk and responsiveness, it resulted that the performances of AI were better in the volatile market, on the other hands, the traditional methodologies ensured stability. However, the interdependence of data and the over-fitting of the model were barriers. DPG reinforcement learning was adopted to estimate a sentiment-aware CVaR-based portfolio optimization framework [29] and used the sentiment scores of FinBERT in the state space to be dynamically assigned. Results produced higher annual return, Sharpe, Omega, and Jensen Alpha versus the various standards, while limitations of accounted to data complexity in calculations and the overall consensus of multi-assets. The relevant article [30] discussed how ML methods are utilized to forecast the financial risk and fine-tune a portfolio. RF, GB, LSTM and Transformer networks were adopted enhanced by structured asset preceding and feature selection schemes and proved higher forecasting precision and Sharpe and Sortino ratios. conversely, it was constrained by the intricacies of calculations and proneness to modulation of market regimes.

### 1.1. Research gaps and motivation

The existing research of capital allocation and portfolio optimization, ongoing advancements through the application of ML, DL and limited DRL approaches. Despite these developments, major research pitfalls on how to effectively address time-varying uncertainty, stability of the learning policy, and risk-sensitive stability of the action selections have not been solved yet. For example, most DRL approaches based on the DQN [27] were facing problems of overestimation bias and unstable value convergence under unpredictable market dynamics, reducing the possibility of reliably stabilizing a stable but increasingly volatile investment portfolio. Similarly, policy-gradient DRL algorithms, such as PPO [29], enhance training stability even further, but continue to have slow adaptation capabilities in response to meaningful regime changes and were sensitive to reward designs.

To overcome these limitations, the proposed DAC-CPPO method facilitates stable policy updating, decreased gradient estimate variance, and convergence-stability. The DRL implementation provides a stable convergence, risk-conscious DRL approach adaptable to dynamic market environments. Consequently, the proposed principle-driven method effectively resolves DRL shortcomings by optimally allocating capital in risky financial markets. The main research contributions of this work are described below,

- Propose a Dynamic Actor-Critic enriched CPPO (DAC-CPPO) model for more adaptive and stable portfolio optimization.
- Obtains risk-aware reward mechanisms that preserve capital in periods of market volatility and uncertainty.
- Provides a means of stabilizing policy updates (using clipping), which diminishes the gradient variance in training and avoids destructive large movements in parameter space.
- Demonstrates superior risk-adjusted performance compared to traditional and standard reinforcement learning portfolio strategies.

## 2. Methodology for optimal capital allocation

The aim of this research is to optimize capital allocation using RL. Intelligent Finance Assets and Massive Yahoo Finance datasets are collected from the publicly available source Kaggle, including historical stock prices, trading volume, and technical indicators. Feature extraction was performed using LDA to enhance model performance. The proposed DAC-CPPO model incorporates dynamic actor-critic with clipped PPO. The overall architecture is depicted in Fig 1.

**Fig 1:**
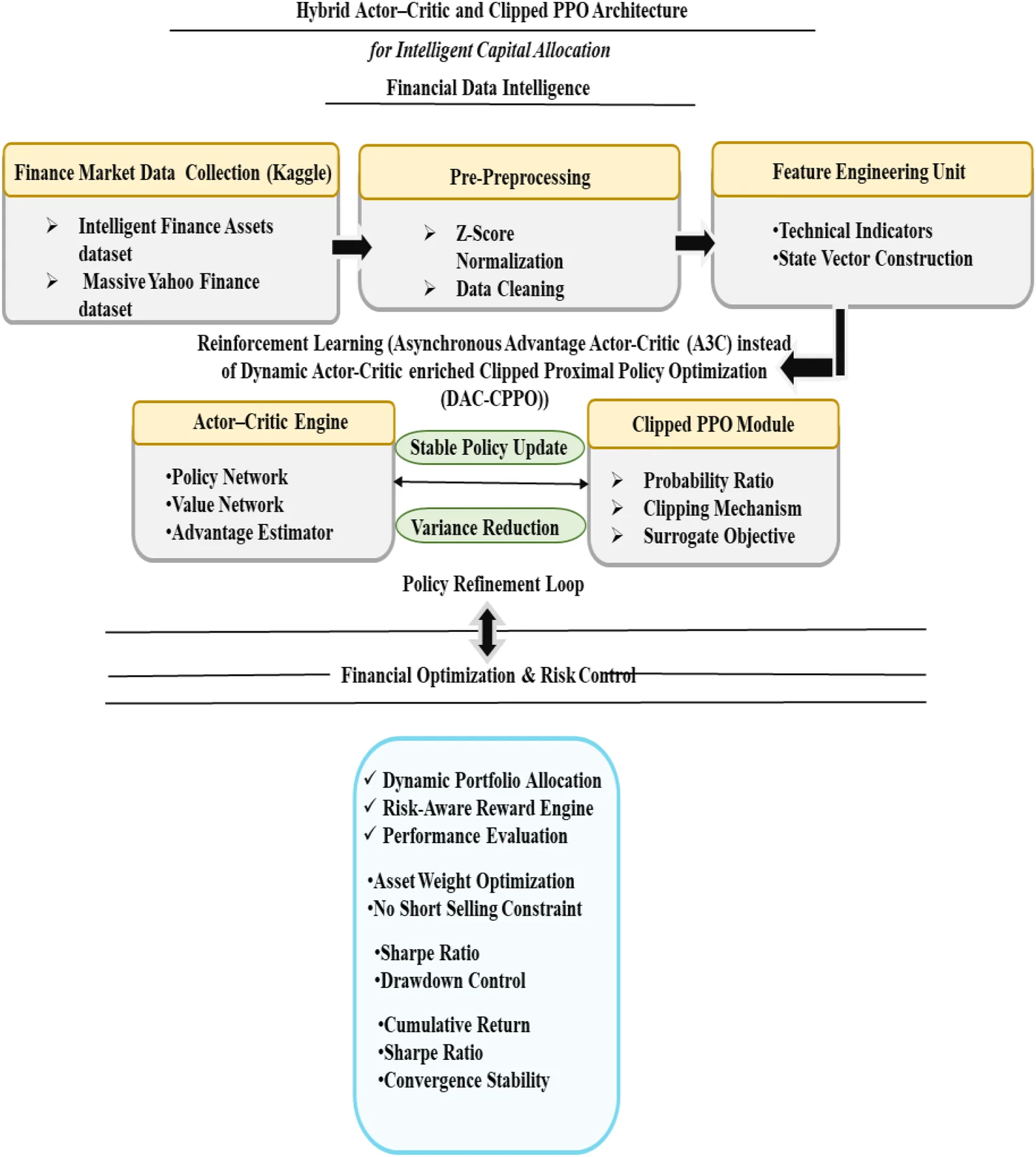
Architecture of the proposed DAC-CPPO model for optimal capital allocation.

### 2.1. Data collection and source description

**Massive Yahoo Finance Dataset (**Yahoo Finance Dataset Historical Stock Market Information of Top 500 Companies). The data at **https://www.kaggle.com/datasets/iveeaten3223times/massive-yahoo-finance-dataset** and **Intelligent Finance Dataset** (Market indicators and capital allocation signals for adaptive portfolio decision) **https://www.kaggle.com/datasets/zara2099/intelligent-finance-assets-dataset**, contains 7000 time-indexed financial records. Both datasets are publicly available Kaggle datasets used for financial analysis and portfolio optimization research purposes. Historical stock market data spanning five years were collected from Yahoo Finance for this dataset. It contains daily records of stock performance measures of the best 500 companies, spanning a period of five years by market capitalization, and the Intelligent Finance Dataset contains fielded financial market data that is intended to be used in classifying an asset and also in intelligent portfolio analysis. It incorporates historical price data, trading, behavior of returns, volatility, and financial risk measures to enable advanced financial modelling and predictive analytics. The dataset is split using a chronological (time-series-based) partitioning strategy, where 80% of the earliest records are used for training, and the remaining 20% of the most recent records are used for testing. To avoid look-ahead and data leakage biases, all financial features employed in training the model have been calculated solely based on historical observations up until the point of prediction. Neither future stock prices, nor returns, nor portfolio results, nor market information have been included at any stage of the feature generation or state representation process. The projection matrix obtained through the LDA transformation has been applied only to the testing set after being trained on the training set. The train-test split is done chronologically to ensure proper preservation of the temporal order of market observations. This provides a realistic assessment in financial forecasting application. It shows the financial account documents to file a portfolio based on the records, feature variables and target categories which is as displayed in Table 1.

**Table 1:** Key features and target variables of financial datasets used in the research.

Dataset	Records	Columns	Features	Target Variable	Target Categories
**Massive Yahoo Finance Dataset**	603k	9	Open, High, Low, Close, MA_5, MA_20, EMA_12, EMA_26, Momentum, Returns, Volatility, MACD, Beta, Sharpe Ratio, Market Sentiment, Liquidity, LDA Features	Capital_Action	Increase, Hold, Decrease
**Intelligent Finance Dataset**	7k	32	Date-Related: Date Price Indicators: Open, High, Low, Close Trading Volume: Volume Corporate Actions: Dividends, Stock Splits Company Info: Company name	Assets (Equity, Bond, Commodity, Forex, Crypto)	Equity, Bond, Commodity, Forex, Crypto

### 2.2. Financial data cleaning and normalization process

Preprocessing involves cleaning data of missing and noisy values and Z-score normalization to set features to a zero mean and unit variance, precluding instabilities and bias in training and enhancing prediction of financial data.

***Data cleaning:*** Data cleaning was performed to correct inaccuracies, missing values and errors in the financial data set before usage in training of the model. In this research, the missing stock prices and anomalous trades with their appropriate imputation methods. Specifically, missing values were handled using linear interpolation for short gaps and forward-filling for sequential missing trading records to maintain temporal continuity. Outlier detection was performed using the Z-score method, where data points with a Z-score greater than ±3 were considered abnormal and treated as outliers. These outliers were either removed or capped, depending on their severity, to ensure stability in model training.

After cleaning, the data set was normalized with Z-score after data clearing and the normalized data was set as input to the model. The normalized data set provide a more accurate and steadier dataset.

***Z-score normalization:*** This process normalizes all the features using a normalization method called as Z-score normalization to get all features on a comparable scale. This makes the features trading volume, stock returns and technical indicators comparable, as well as improving the stability of the Actor-Critic network by reducing the effects of extreme values. The normalization can be described as follows by using equation (1).


$$\begin{array}{c} z_{i}=\frac{x_{i}-\mu }{\sigma } \end{array}$$ (1)

Where $x_{i}$ is the raw value of the data set, μ is the mean value, $z_{i}$ is the standardized value, and σ is the standard deviation of the feature.

The output from this preprocessing, including cleaned data and normalized features, is a refined dataset that enhances LDA extraction by improving feature consistency, reducing noise, and ensuring more accurate portfolio optimization. Furthermore, the final processed dataset is structured in chronological order before model training to prevent information leakage in financial time-series forecasting.

### 2.3. Linear Discriminant Analysis (LDA) based financial feature extraction for portfolio optimization

This process uses the preprocessed data to obtain discriminative features, diminish noise, and make LDA effective in stabilizing the DAC training to maximize portfolio performance. Being a supervised dimensionality reduction method, LDA projects the high-dimensional data into a lower-dimensional space by maximizing the amount of variance between classes and minimizing the amount of variance within a class through the best projections. An intelligent portfolio allocation model uses the LDA to minimize co-relationship among financial indicators in prices, MACD, volatility, volume, and returns. This lowers noise, the risk of overfitting, improves the patterns of discriminative markets, and stabilizes the training of DAC. Consequently, LDA strengthens state representation, supporting stable and risk-aware portfolio optimization.

Equation (2) illustrates the Maximized discriminative financial features, improving DAC learning stability performance. It is used to reduce data dimensions by maximizing class separation and minimizing similarities, improving feature discrimination for portfolio allocation.


$$\begin{array}{c} X_{opt}=\begin{array}{c} argmax\\ \omega \end{array}\frac{\left|X^{T}T_{D}X\right|}{\left|X^{T}T_{\upsilon }X\right|}=[\omega_{\text{1}},\omega_{\text{2}},..,\omega_{K}] \end{array}$$ (2)

Where $X_{opt}$ is represented as an Optimal projection matrix, $[\omega_{\text{1}},\omega_{\text{2}},..,\omega_{K}]$ is the projection vectors, X is the financial data, $X^{T}$ is the transpose of financial data, reduced dimension size is denoted as k,$T_{D}$ denotes the between-class scatter matrix, $T_{U}$ denotes the within-class scatter matrix, |.| is mentioned as determinant value, $\begin{array}{c} argmax\\ \omega \end{array}$ denotes the argument of the maximum function, and ω is the feature vector. $T_{D}$ Between-class scatter is mathematically represented in Equation (3), which determines the proportion of total wealth to allocate to each asset, optimizing for expected return and risk.


$$\begin{array}{c} T_{D}=\sum_{l=\text{1}}^{b}N_{l}\left(\mu_{l}-\mu \right){(\mu_{l}-\mu )}^{T} \end{array}$$ (3)

Reduces intra-class financial noise, improving risk-adjusted feature consistency significantly. It is mathematically described in equation (4). In Equation 4, the market's current condition and the returns expected from assets guide portfolio decisions based on past and present data.


$$\begin{array}{c} T_{U}=\sum_{l=\text{1}}^{b}\sum_{wv\in W_{l}}(w_{v}-\mu_{l}){(w_{v}-\mu_{l})}^{T} \end{array}$$ (4)

b is the Number of classes,$T_{U}$ is the within-class scatter, $N_{l}$ is the number of classes, $\mu_{l}$ is the class mean vector, μ is the global mean vector, $\left(\mu_{l}-\mu \right)$ is the mean difference vector, $\sum_{l=\text{1}}^{b}$ denoted the summation of overall response,T is the Transpose operator, and the Class sample set and Individual sample vector are denoted as ${(w}_{l}andw_{v})$, $w_{v}-\mu_{l}$ is the sample deviation and Eigenvalue importance score.

The projection vectors $w_{k}$ are obtained by solving the generalized eigenvalue problem. Equation (5) calculates the portfolio's performance (reward) based on returns and risk, such as the Sharpe ratio, balancing profitability and volatility.


$$\begin{array}{c} T_{D}w_{k}=\lambda_{k}T_{U}w_{k} \end{array}$$ (5)

Keigenvectors corresponding to the largest eigenvalues $\lambda_{k}$eigenvectors corresponding to the largest eigenvalues, $w_{k}$is the Optimal basis vector, mathematically presented in equation (6). It produces compact state inputs, accelerating Actor–Critic convergence performance. Adjusts the policy (portfolio weights) based on reward feedback, promoting better decision-making in future actions for optimized returns.


$$\begin{array}{c} z^{t}=W^{T}x_{t} \end{array}$$ (6)

$z^{t}$is the reduced state vector, $W^{T}$is the projection matrix transpose, and $x_{t}$ is an original feature vector. LDA gives concise, inherently discriminative financial feature vectors, removing noise and redundant information. This transformation ensures compact, noise-reduced, and highly discriminative state representations, which are then passed to the DAC-CPPO model to improve convergence stability, accelerate learning, and enhance risk-aware portfolio allocation performance.

### 2.4. DAC-CPPO for intelligent portfolio optimization

A reinforcement learning framework by combining the use of DAC and CPPO in portfolio investment was designed for intelligent capital allocation. The Actor Critic mechanism allowed for flexible policy learning and value estimation in the dynamic non-stationary market. CPPO helped to provide stable convergence of policy learning and prevent over-rebalancing and reallocation of the portfolio by clipping at an ideal probability ratio. This framework achieved significant improvement in convergence stability, Sharpe ratio, long-term risk adjusted capital allocation and avoided over-rebalancing of the portfolio that causes severe drawdown risks.

#### 2.4.1. DAC model for multiple agents interacting with market scenarios to update shared network.

DAC is deep RL algorithm, compose two neural nets. One is Actor Network, which learns a policy $\mu (a_{t}|s_{t})$, the action that enhances the choice (invest every county to certain proportion of total capital) according to the state of market body. Another is Critic Network, which evaluates the action taken by approximating the value function $V^{\mu }(T_{s})$, function value to giving the actor a useful guidance to make a better decision. The main feature of DAC is that it is an asynchronous parallel training, which means that the agents can adapt to various market conditions at the same time and modify one global network of policies. It also results in more reliable stability as well as a faster rate convergence. It can be applied to make flexible financial decisions, generate continuous policy, give a considerable attention to the detailed capital allocation, and practical-flexible adaptation the ability of market environment under the support from DAC.

The state space $s_{t}$ represents market conditions, including stock prices, trading volume, and volatility indicators. The action space defines portfolio allocation weights, while the reward $T_{Reward}$ measures risk-adjusted returns, optimizing performance through return–volatility trade-offs.

Processes normalized market states, such as prices, MACD, volatility, and portfolio weights, are in Processes in the DAC Input Layer to learn smoothly on. Hidden layers are the further developed layers that extract time and latent market characteristics and highlight the significant indicators in the predictive allocation. To obtain risk-sensitive portfolio weighting based on a softmaxactivator, Actor Network is applied, which optimizes a sum-to-one allocation that is stable. Critic Network: Reckon expected returns with TD error and benefit functions to evaluate values. Output Layer generates an allocation output of CPPO to stabilize an allocation output, to avoid extreme updates used to achieve adaptive, risk-aware, and high Sharpe ratio performance in dynamic financial markets.

Prevents large policy updates by clipping probability ratios, ensuring stable training. It is mathematically represented in equation (7). Actor adjusts portfolio weights; critic estimates value, refining both networks efficiently. That maximizes cumulative discounted reward; this formula is presented in equation (8).


$$\begin{array}{c} PolicyFunction=\mu (a_{t}|s_{t}) \end{array}$$ (7)

$$\begin{array}{c} T_{g}=T_{Reward+\text{1}}+{\gamma T}_{Reward+\text{2}}+\gamma^{\text{2}}T_{Reward+\text{3}}^{\text{2}}\ldots \ldots \ldots \ldots . \end{array}$$ (8)

Allocation action is represented as $a_{t},$ $s_{t}$ is the market state. $T_{g}$ denotes the long-term portfolio performance,γ is the discount factor (0–1), $T_{Reward}$is an immediate reward, and T is mentioned as the Time step.Reward+1 is the next-period reward, Reward+2 is denoted as the future portfolio reward, and Reward+3 is the subsequent financial reward; $\gamma^{\text{2}}$ is the future reward discount.

Evaluates how much better an action is compared to the average action, which is represented in equations (9) and (10). It updates policy parameters to increase the probability of better allocation decisions.


$$\begin{array}{c} \nabla_{\theta a}\text{log}\mu (a_{t}|s_{t};\theta_{a}).Adv(a_{t},s_{t}) \end{array}$$ (9)

$$\begin{array}{c} Adv\left(a_{t},s_{t}\right)=Q\left(a_{t},s_{t}\right)-V(s_{t}) \end{array}$$ (10)

$\nabla_{\theta a}$ is the policy gradient, $Adv(a_{t},s_{t})$ is denoted as an advantage value,$Q\left(a_{t},s_{t}\right)$ is an action value, μ is denoted as an action probability policy, and $V(s_{t})$ is the state value.

The Critic measures the quality and effectiveness of the Actor’s decisions. State value functions and State-action value functions are represented in equations (11) and (12). These functions evaluate whether the allocation decision improves expected financial return.


$$\begin{array}{c} V^{\mu }\left(s_{t}\right)=E_{\mu }[T_{g},s_{t}] \end{array}$$ (11)

$$\begin{array}{c} Q^{\mu }\left(a_{t},s_{t}\right)=E_{\mu }[T_{g},s_{t},a_{t}] \end{array}$$ (12)

$V^{\mu }\left(s_{t}\right)$ is an expected state return, $Q^{\mu }\left(a_{t},s_{t}\right)$ is an expected action return, and $E_{\mu }\left(.\right)$ is a Policy expectation. TD Error is a mathematical derivation presented in equation (13). This error helps refine risk-adjusted return estimation during training.


$$\begin{array}{c} \delta_{T}=T_{Reward}+\gamma V\left(s_{t+\text{1}}\right)-V(s_{t}) \end{array}$$ (13)

Where $\delta_{T}$ denotes the Temporal Difference (TD) error, $T_{Reward}$ represents the immediate reward, γ is the discount factor for future rewards, $V\left(s_{t+\text{1}}\right)andV(s_{t})$ denote the value functions of the next and current financial states, respectively, while $s_{t+\text{1}}ands_{t}$represent the next and current intelligent finance states used in portfolio optimization learning. The Actor-Critic model is a dynamically optimizing model of portfolio weights that dynamically adjusts to non-stationary markets, accelerates convergence through parallel learning, trades off exploration and exploitation, and can reduce policy variance through advantage estimation to achieve stable performance. The DAC model improved on earlier models in the cumulative returns, risk-adjusted stability, robustness in volatile market conditions, convergence rate, and decisions on capital rebalancing, making it more adaptive toward a more realistic economic behavior.

Fig 2 demonstrates the DAC model of dynamic portfolio optimization. In this model, the Actor-Critic network is a combination of global networks, which provide information about the portfolio allocation based on parallel market simulation agents in various market conditions, such as volatility and stability. Based on TD error, the shared parameters are updated, and continuous strategy and assessment function updates are made to better allocate assets with time.

**Fig 2:**
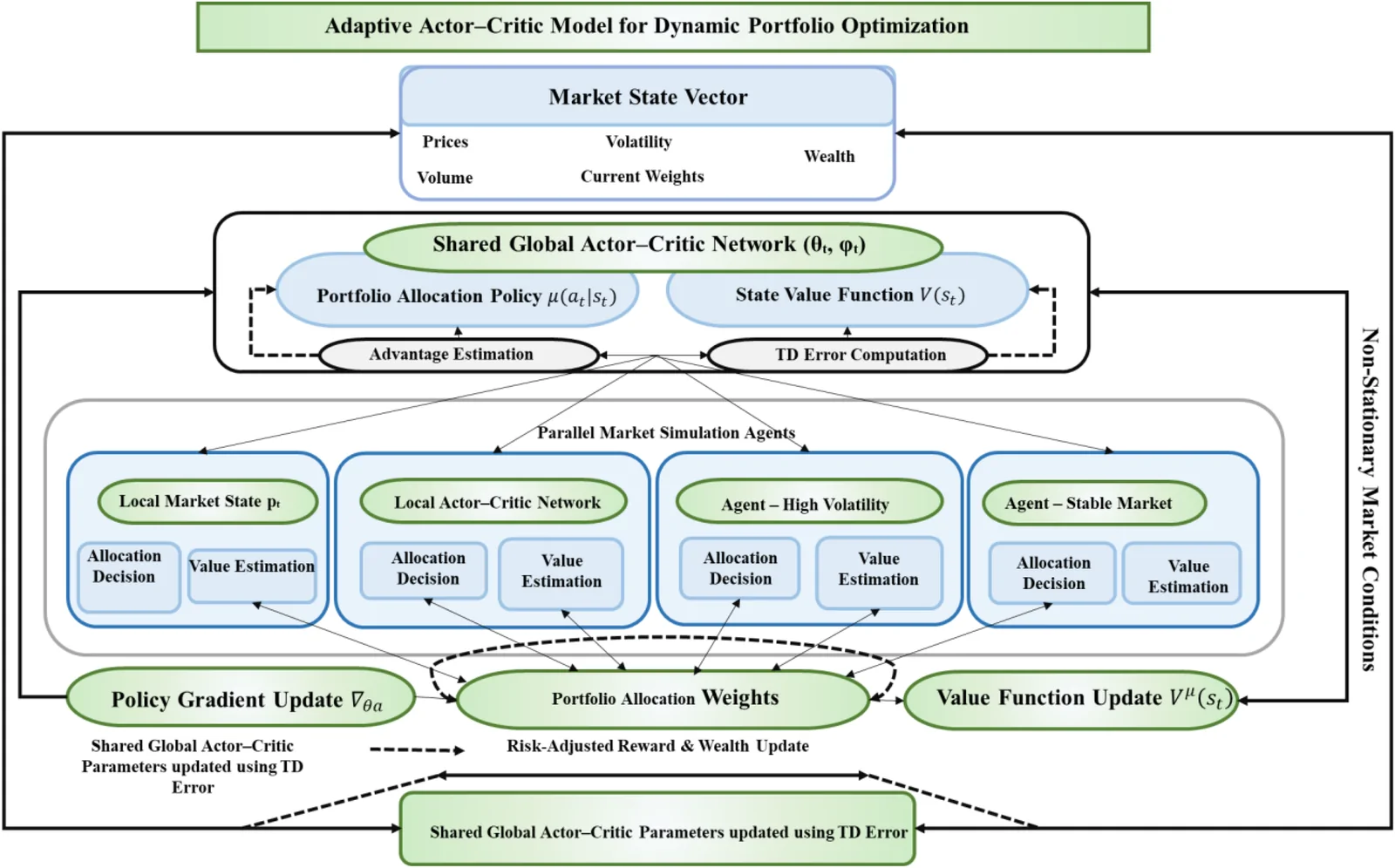
DAC model for multiple agents interacting with market scenarios and shared network updates.

#### 2.4.2. CPPO for stabilizing and improving DAC learning performance.

CPPO is used to stabilize portfolio allocation changes in volatile financial market conditions. It controls portfolio adjustments by limiting the magnitude of policy updates during DAC training. The alleviate the overactive portfolio turnover, risk exposure due to instability, and convergence of non-stationary financial time series. In this proposed model, CPPO is employed as a policy stabilization layer on the top of the DAC policy, to prevent the sudden and overly aggressive real portfolio rebalancing decisions when the asynchronous actor policies updates. As a policy gradient reinforcement learning algorithm, traditional PPO maximizes a surrogate objective function derived from the probability ratio between the new policy and the current policy to regulate the training process. But from the perspective of a financial portfolio rebalancing problem, even PPO, the state-of-art contemporary algorithm, still introduces massive policy shifts under extreme market turbulence, which may destabilize the portfolio reallocation and adds financial risk to an impractical level. To comprehend this deficiency, the proposed CPPO incorporates a constrained clipping process, adapted to financial portfolio rebalancing task specifically, to maintain the weight updates in proportion to a pre-specified threshold bound when market variance becomes high. CPPO contorts the probability ratio within a preaged spectrum, avoiding any abrupt policy shifts or unstable rebalancing decisions, enhances the risk-adjusted returns, improves the portfolio's risk exposure, and bears better robustness in a finance market.

Maximizes the total long-term risk-adjusted cumulative returns with respect to the portfolio when the financial markets are in the presence of dynamic and unstable conditions. The form of the analytical expression is shown in Equation (14).


$$\begin{array}{c} V\left(s\right)=\;E\left[\sum_{t=\text{0}}^{\infty }\gamma^{t}r(a_{t},s_{t})\right] \end{array}$$ (14)

This ensures the model does not optimize only short-term profit but focuses on sustainable capital growth. It balances immediate reward and future financial stability using discount factor γ.V(s) is the state value, E is denoted as the Expectation operator, $s_{t}$ is the Market state (price, MACD, volatility), $\sum_{t=\text{0}}^{\infty }$is the summation of the overall response tto∞, $a_{t}$ is the Portfolio allocation weights, $r(a_{t},s_{t})$ is the Risk-adjusted return, and the time step is represented as t.

Measures allocation shift intensity between previous and updated portfolio decision policies. The formulation is presented in Equation (15).


$$\begin{array}{c} r_{t}\left(\theta \right)=\frac{\pi_{\theta }(a_{t}|s_{t})}{\pi_{\theta_{old}}(a_{t}|s_{t})} \end{array}$$ (15)

The ratio of probabilities is defined as $r_{t}\left(\theta \right)$, where $\pi_{\theta }$ is the active policy, $\pi_{\theta_{old}}$is the Previous policy, and (θ) is the policy parameters. Improves portfolio allocation toward higher advantage actions but may cause unstable weight updates. The formulation is presented in Equation (16).


$$\begin{array}{c} L_{CPI}\left(\theta \right)=\hat{E_{t}}\left[r_{t}\left(\theta \right)\hat{A_{t}}\right] \end{array}$$ (16)

$L_{CPI}\left(\theta \right)$ is the Surrogate objective, $\hat{E_{t}}$ is the batch expectation, and $\hat{A_{t}}$ is an Advantage estimate. Restricts excessive portfolio weight updates to maintain financial stability under volatile and uncertain market environments. The detailed mathematical representation is given in Equation (17).


$$\begin{array}{c} L_{CLPI}\left(\theta \right)=\hat{E_{t}}\left[\text{min}(r_{t}(\theta ){\hat{A}}_{t},Clip(r_{t}\left(\theta \right),\text{1}-\in ,\text{1}+\in ){\hat{A}}_{t})\right] \end{array}$$ (17)

∈ is the small threshold (e.g., 0.2), Stable convergence, reduced risk spikes, controlled rebalancing, and improved Sharpe ratio performance in practical evaluation. The Limits probability ratio range is mathematically represented in equation (18).


$$\begin{array}{c} Clip(r_{t}\left(\theta \right),\text{1}-\in ,\text{1}+\in ) \end{array}$$ (18)

The Clipped objective is denoted as $L_{CLPI}\left(\theta \right)$,$\hat{E_{t}}$ is the Batch average, $r_{t}\left(\theta \right)$ is the Probability ratio, ${\hat{A}}_{t}$ is an advantage value, ∈ is the Clipping threshold, min() is the Conservative selection, andclip() is the denoted Ratio limiter. CPPO ensures controlled portfolio rebalancing by limiting excessive policy updates, reducing drawdown risk, improving learning stability, enhancing Sharpe ratio performance, and enabling adaptive capital allocation under non-stationary financial market conditions.

Fig 3 demonstrates the process of clipped PPO; the input data goes through the input embedding layer, processed by the policy and value networks. The actor decides on portfolio allocations, and the critic evaluates them based on past experience. CPPO ensures stable updates and improves the risk-adjusted return, ensuring that the model doesn't make drastic changes that could lead to instability.

**Fig 3:**
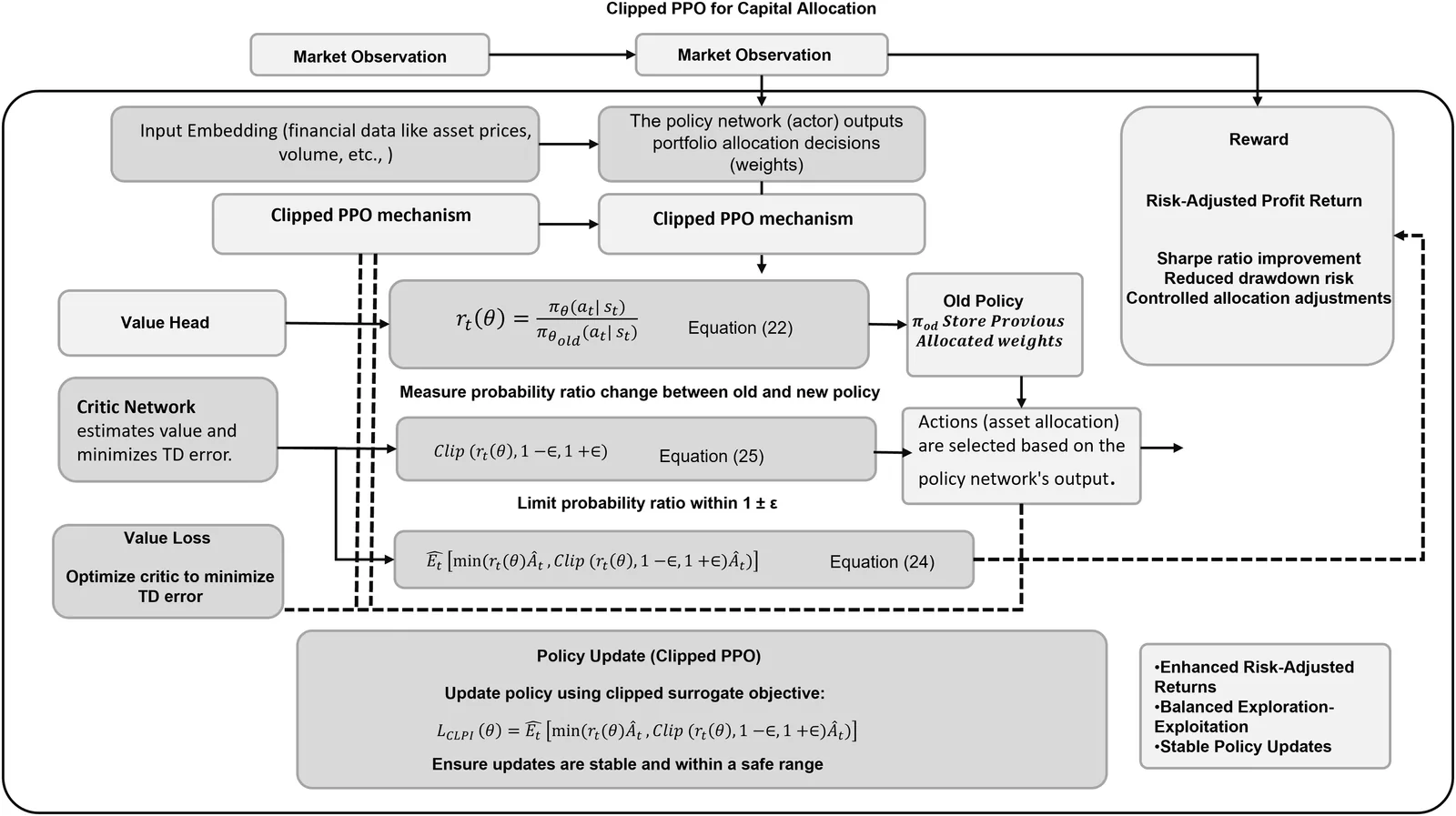
CPPO process for stable portfolio optimization and risk-adjusted return calculation.

An intelligent portfolio allocation leverages a hybrid DAC-CPPO model that integrates DAC with CPPO for stable, risk-aware capital allocation. The Actor network dynamically generates portfolio weights, while the Critic estimates expected long-term returns, using temporal-difference errors and advantage functions to guide learning. DAC’s asynchronous parallel training improves convergence, balances exploration–exploitation, and adapts to non-stationary market conditions. CPPO constrains policy updates by limiting probability ratios between new and old policies, preventing excessive reallocations, controlling drawdown, and enhancing the Sharpe ratio performance. Combined, DAC-CPPO ensures adaptive, stable, and risk-adjusted portfolio management, achieving higher cumulative returns, reduced volatility exposure, and robust long-term capital growth under dynamic financial environments. Compared to traditional DRL portfolio methods such as standard Actor-Critic, DDPG, and PPO-based approaches, which often suffer from unstable policy updates, high variance, and sensitivity to market noise, the proposed DAC-CPPO model overcomes these limitations by introducing asynchronous multi-agent learning with clipped policy optimization, resulting in improved stability and better risk-adjusted performance.

An intelligent portfolio allocation framework is developed using the proposed DAC-CPPO architecture, which integrates DAC with CPPO to ensure stable and risk-aware capital allocation. Multiple worker agents interact with parallel financial environments and asynchronously update the global Actor–Critic networks. The Actor network generates allocation actions that determine portfolio weights, while the Critic estimates the value function $V(s_{t})$ to evaluate expected long-term returns. DAC layers normalize market states, extract latent features in hidden layers, generate risk-aware portfolio weights via the Actor Network, estimate returns with the Critic, and stabilize updates with CPPO. Advantage functions computed from temporal-difference errors guide policy learning. DAC’s asynchronous training improves convergence speed and enhances exploration in non-stationary financial markets. The CPPO objective constrains policy updates using probability-ratio clipping, preventing excessive portfolio reallocations and reducing drawdown risk. Unlike conventional DRL methods that directly update policies and may lead to unstable portfolio swings under volatile conditions, the proposed CPPO constraint ensures controlled and smooth rebalancing of assets. In turn, the DAC-CPPO model attains balanced weights of portfolio, better Sharpe ratio performance, lower volatility exposure, and strong long-term capital growth within fluctuating financial conditions. Fig 4 illustrates how the DAC-CPPO model is integrated to optimize a portfolio. The input environments (Env) are the financial market data and macroeconomic indicators, whereby the worker agents are subjected to varied market conditions. The agents produce portfolio weight, and the global actor-critic network optimizes the model with the CPPO constraint. The result is a stable allocation of risk-aware portfolio to market conditions.

**Fig 4:**
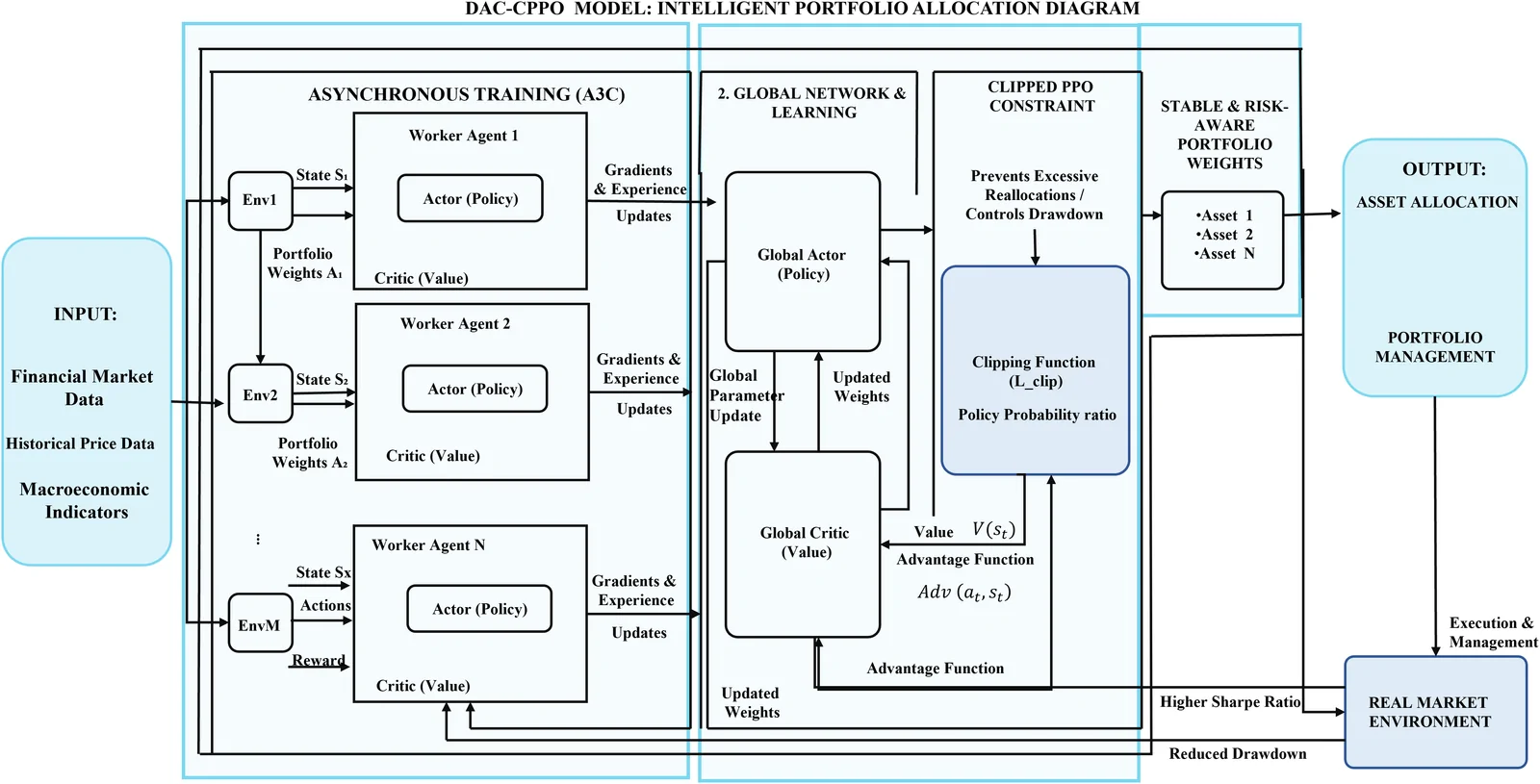
Proposed DAC-CPPO integration for stable portfolio optimization and risk management.

**Algorithm 1** demonstrates the Pseudocode for DAC-CPPO, the process of asynchronous Actor–Critic networks with CPPO to dynamically generate portfolio weights, optimizing risk-adjusted returns while controlling drawdowns and ensuring stable, adaptive capital allocation.

***Algorithm 1: DAC-CPPO Reinforcement Learning for Portfolio Allocation***

*Input:*

 *Market states: s_t = [δ_t, w_t, l_t, x_t], Number of assets: D, Discount factor: γ ∈ (0,1)*

 *Transaction cost function: c(a_t, s_t), Learning rates: α_actor, α_critic, Clipping threshold: ε (e.g., 0.2), Investment horizon: T*

*Initialize Networks:*

 *- Actor μ(a_t|s_t; θ_a) with dense + LSTM layers*

 *- Critic V(s_t; θ_c) with dense + LSTM layers*

 *- Initialize global parameters and asynchronous workers*

*for t = 0 to T do*

*Actor chooses allocation weights*

*a_t ~ μ(a_t|s_t; θ_a)*

*Compute next-period wealth*

x_(t + 1) = x_t * (w_t^T * δ_(t + 1) – c(a_t, s_t))

*Compute risk-adjusted reward (Sharpe ratio)*

*r_t = μ_t / σ_t*

*Observe next state s_(t + 1)*

*Critic update: compute TD-error*

θ_c ← θ_c + α_critic * δ_t * ∇*_θc V(s_t)*

*Actor update using the Advantage function*

*Adv(a_t, s_t) = Q(a_t, s_t) – V(s_t)*

θ_a ← θ_a + α_actor * ∇*_θ L_CLIP(θ)*

*Update s_t ← s_(t + 1)*

*end for*

*end for*

*Return:*

 Optimal policy π*(a_t|s_t) = argmax_πθ J(θ)

 *Portfolio wealth trajectory x_t*

Table 2 outlines key hyperparameters, their descriptions, and recommended value ranges to optimize training stability, exploration, and portfolio optimization performance in RL models.

**Table 2:** Hyperparameters for DAC-CPPO model training.

Hyperparameter	Description	Recommended Range	Experimental Value
Learning Rate (Actor)	Step size for actor policy updates	0.0001–0.003	0.0003
Learning Rate (Critic)	Step size for critic value updates	0.0005–0.002	0.001
Discount Factor (γ)	Weight assigned to future rewards	0.95–0.999	0.99
Clipping Range (ε)	PPO clipping threshold for stable policy updates	0.1–0.3	0.2
Batch Size	Number of training samples per update	32–128	64
Replay / Memory Size	Experience buffer capacity	20,000–100,000 transitions	50,000 transitions
Number of Parallel Agents	Asynchronous DAC training agents	4–16	8
Entropy Coefficient	Exploration regularization factor	0.005–0.02	0.01
Gradient Norm Clip	Maximum gradient norm for training stability	0.3–1.0	0.5
Training Episodes	Total episodes for model convergence	20,000–100,000	50,000 episodes

## 3. Result and discussion

The experimental setup for the intelligent portfolio optimization model was realized on a workstation with an Intel i7 CPU and 32 GB of RAM. The proposed DAC-CPPO framework is trained on a conventional CPU-based platform without GPU acceleration, which shows that the framework can be trained on conventional CPU-based platforms. In all experiments, the asynchronous multi-agent reinforcement learning framework used 8 parallel agents to achieve a compromise between computational efficiency and convergence stability. The model proposed for DAC-CPPO is implemented in Python 3.10. To assess the convergence stability, risk-adjusted return, improvement in Sharpe ratio and adaptive portfolio allocation performance in dynamic financial markets, historical financial datasets were split into a group with 80% of the most recent data (the training set) and 20% of the least recent data (the test set).

### 3.1. Intelligent portfolio allocation process

The proposed model of architecture of the intelligent portfolio allocation process, DAC-CPPO, is graphically visualized in Fig 5. It is a system that processes practical financial market data, performs state analysis and risk evaluation, and a hybrid Actor-Critic with CPPO engine is used to dynamically optimize the portfolio allocation in the non-stationary market conditions. Suggested DAC-CPPO optimization model of the dynamic portfolio and risk-sensitive allocation. It combines real-time market information (prices, volumes, volatility) and macroeconomic data to make informed portfolio choices. The DAC CPPO engine makes adjustments in the portfolio weights with feedback from the Performance Monitor and Risk Evaluation Engine. The Market State Analyzer identifies the trend and regime change, whereas the Dynamic Portfolio Allocator checks the real-time adjustments, giving the optimal use of assets under the risk.

**Fig 5:**
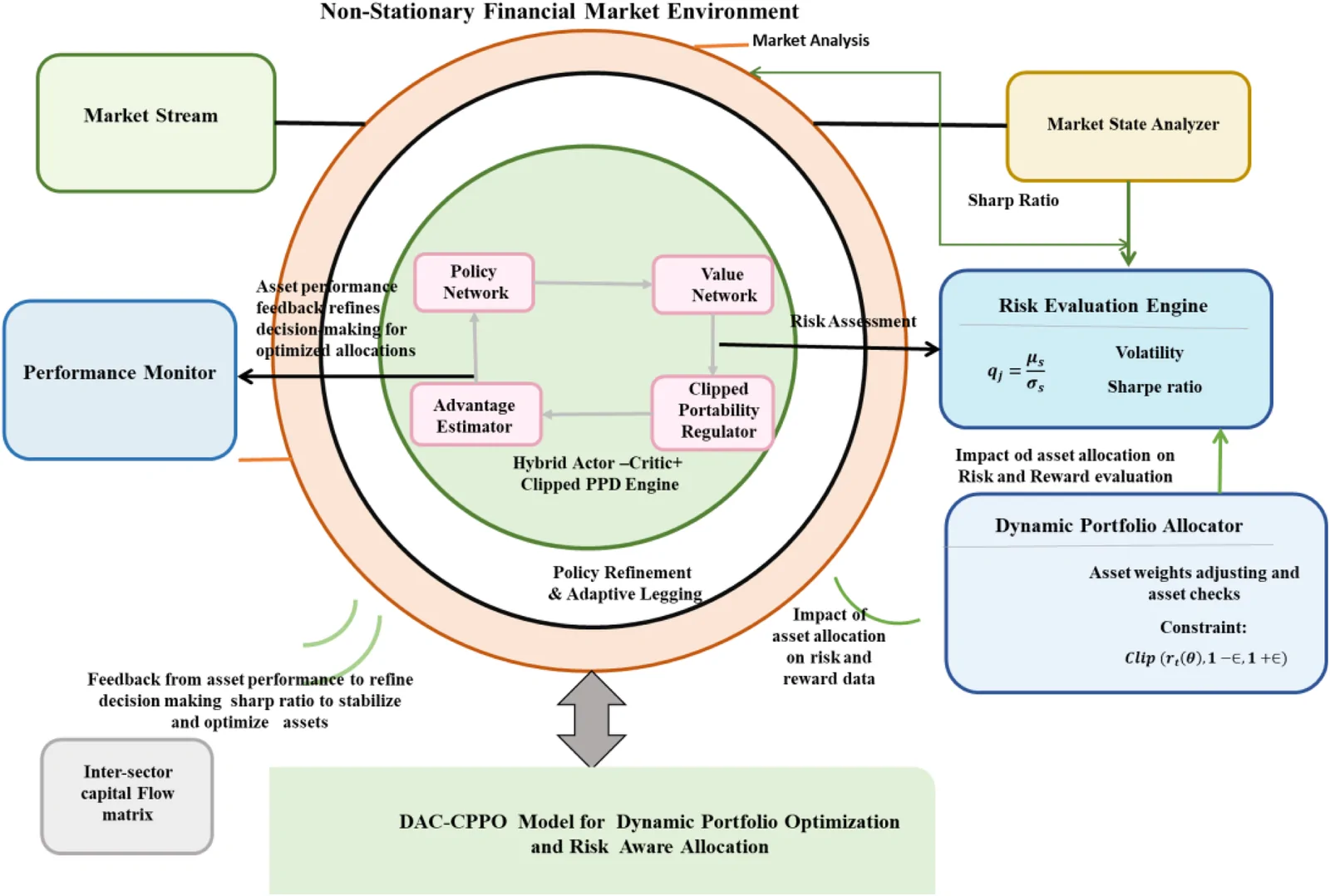
DAC-CPPO model for dynamic portfolio optimization and risk-aware allocation.

The experimental approach used for the intelligent portfolio optimization model involved Intel i7 CPU with 32 GB RAM to achieve fast deep reinforcement learning model training. The proposed model DAC-CPPO was developed with the help of Python 3.10 programming language. First, historical datasets related to financial markets were split into training and testing datasets based on an 80:20 chronological division method to keep the order of observations from the real world. In addition, 5-fold expanding window time series cross-validation technique was applied to further verify the robustness and generalization of the model. In the process of applying this cross-validation scheme, the model was trained with the help of all historical observations up to a certain time point and tested on subsequent unseen period of time. As a result, the size of training window is increased after every iteration. In this way, the presented validation process ensures that there is no look-ahead bias present in the model, and a realistic estimation of convergence, risk-adjusted return, Sharpe ratio, and portfolio allocation can be achieved.

Fig 6 visualizes the distribution of normalized feature values of the different features of financial markets, where the lowest value is + 1.0 (high) and the highest value is −1.0 (low). The characteristics, including the Open Price, Simple Return, and Signal Line of the MACD, are rated, and the values of the various characteristics are represented by various colors. This visualization assists in acquiring knowledge about the spread and the variety of each of the features in the dataset, assisting in the selection of features to be used as models.

**Fig 6:**
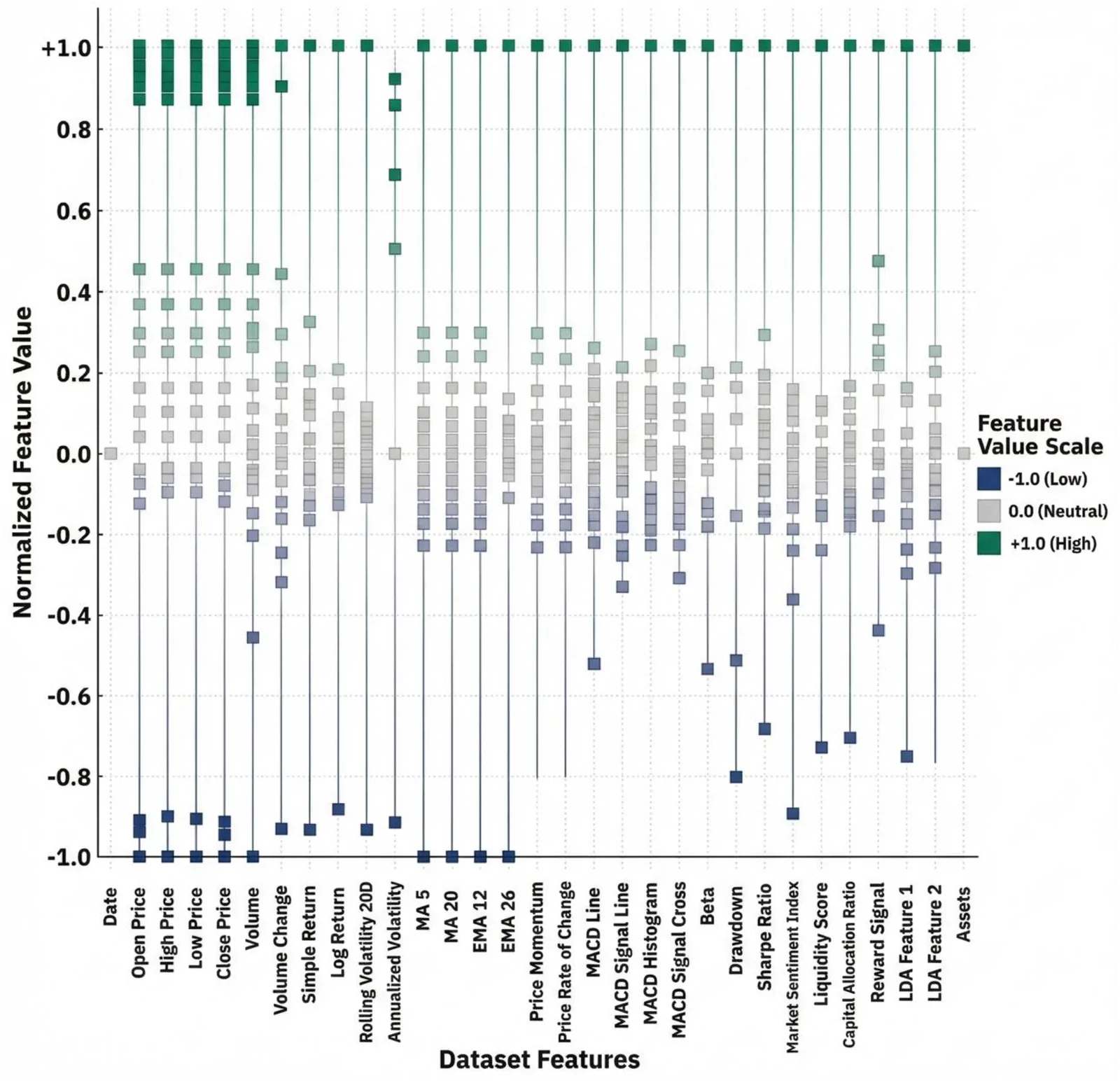
Financial market feature distribution visualization with normalized feature value scales.

Fig 7(a-b) illustrates the cumulative returns and drawdown of DAC-CPPO, DAC, PPO, and a benchmark model on 250 trading days. Fig 7(a) represents the cumulative returns of various models in 250 trading days. DAC-CPPO has an upward trend of returns, and it surpasses the DAC, PPO, and benchmark models. The Characteristics of the shaded area around the DAC-CPPO curve show the variability in the returns of the model, but it is the highest across the board, and this indicates the high level of effectiveness in portfolio management. Fig 7(b), a drawdown plot, puts the risk profile of DAC-CPPO into the limelight. This keeps DAC-CPPO at a minimum drawdown, unlike DAC and PPO, which can be near the zero point. This proves its capability to have minimal losses in times of market crashes; thus, it is more resilient than other models, especially in volatile trading times.

**Fig 7:**
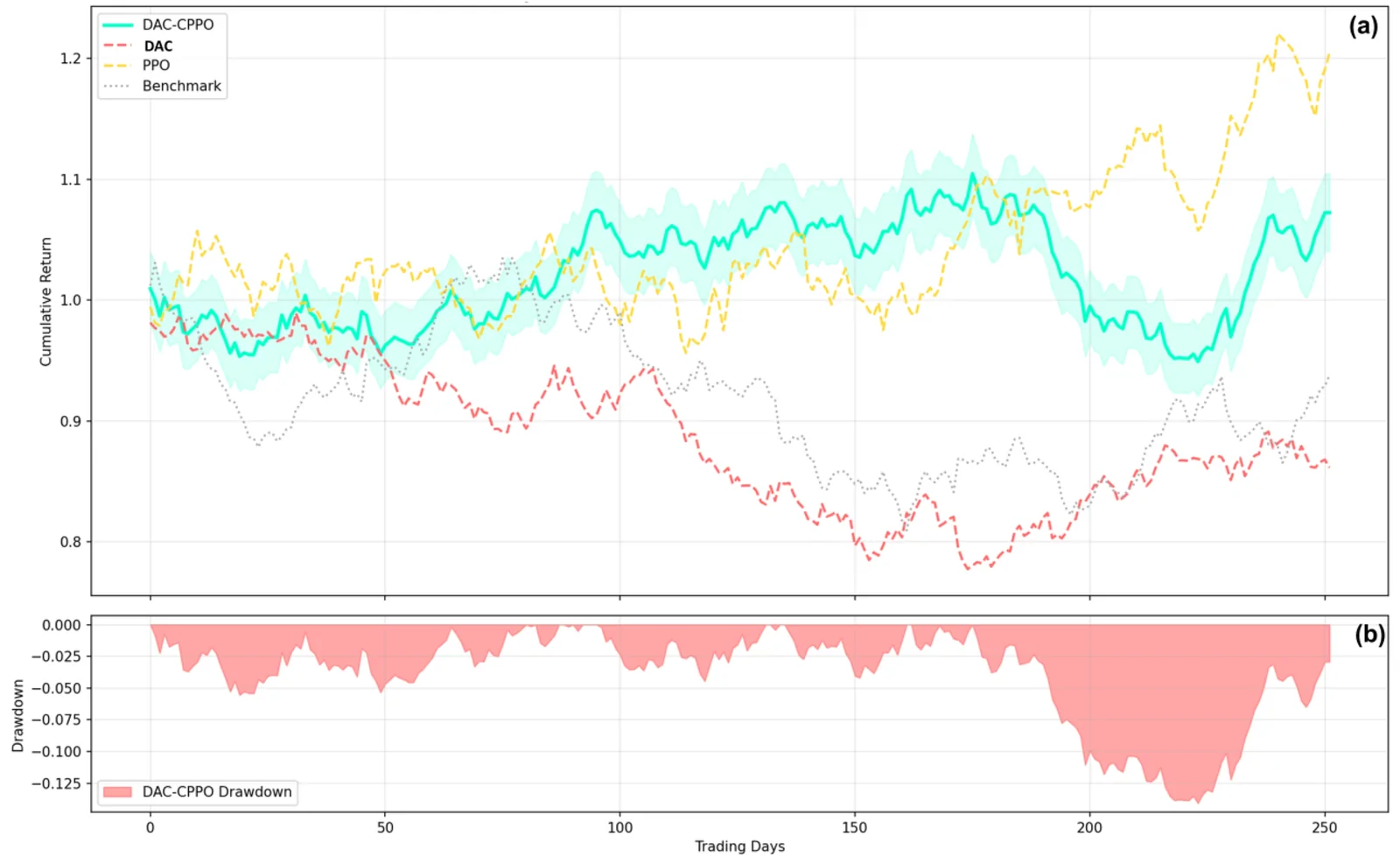
Graphical Representation of the (a) Cumulative Return Comparison: DAC-CPPO outperforms other models in cumulative return (b) DAC-CPPO maintains lower drawdown compared to alternatives.

Fig 8 gives a heatmap of the monthly Sharpe ratios of five models in relation to January to December. At deeper red colors, a higher Sharpe ratio is denoted. The DAC-CPPO model shows consistently good results of 2.05 in July, 2.16 in October, and 1.95 in September, which reflects better performance with respect to the risk-adjusted returns. By comparison, LSTM has its highest value of 2.17 in May but less stability in other months, whereas CPPO and DAC range between 0.85 and 1.69, which indicates mediocre performance. SVM also has significant variation, such as 2.11 in March and only 0.91 in December. On the whole, the steadiest and the highest Sharpe ratios all through the year are shown in DAC-CPPO and reflect its better ability to optimize the portfolio.

**Fig 8:**
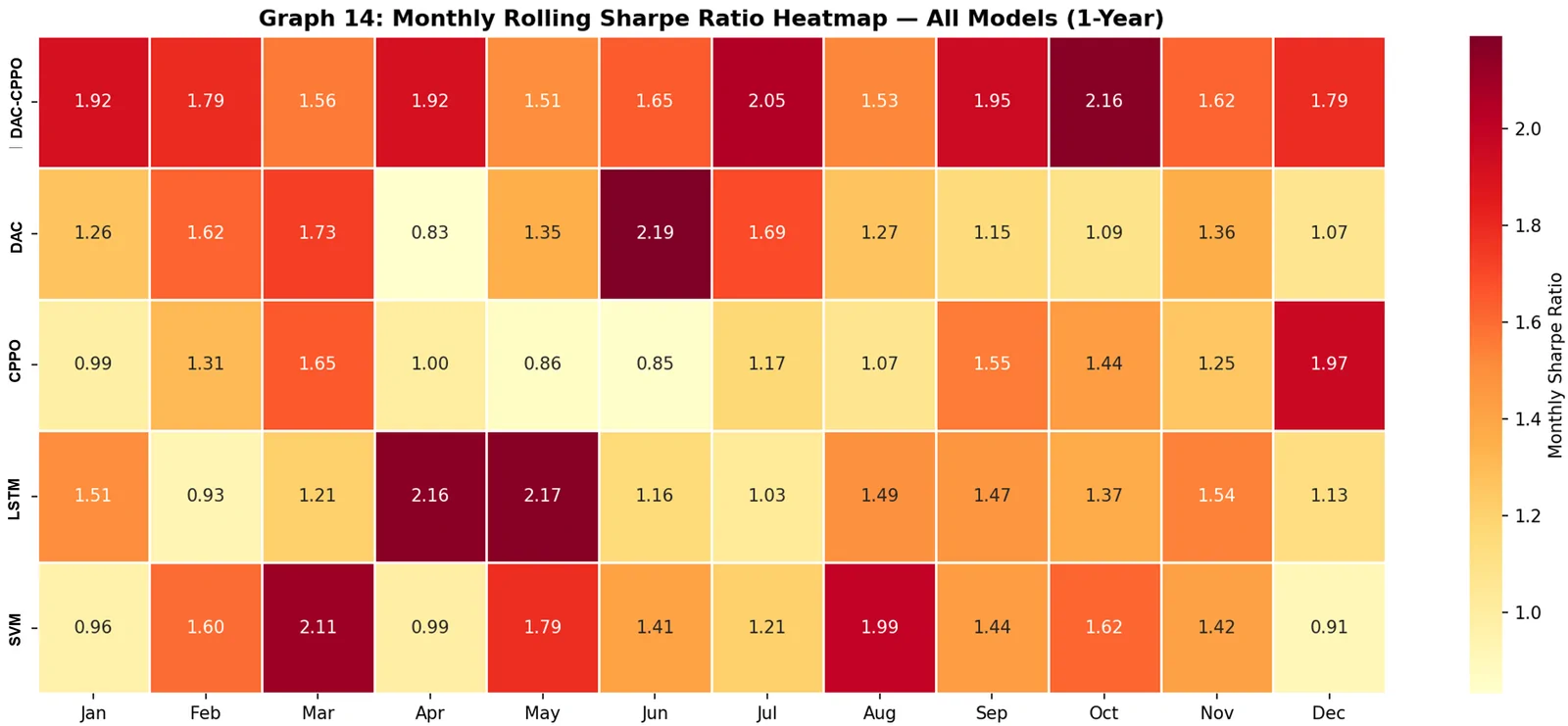
Monthly sharpe ratio comparison across models (DAC-CPPO, DAC, PPO, LSTM, SVM).

The role played by various factors in the ultimate Sharpe ratio of the DAC-CPPO model is shown in Fig 9. The greatest contribution goes to the Base Return of +0.80, then +0.25 of Risk Management. Sharpe ratio is gradually pushed up by using other parts like DAC Bonus, PPO Clip, and LDA Features. The efficiency of the model is proved by the last Sharpe ratio being 1.91.

**Fig 9:**
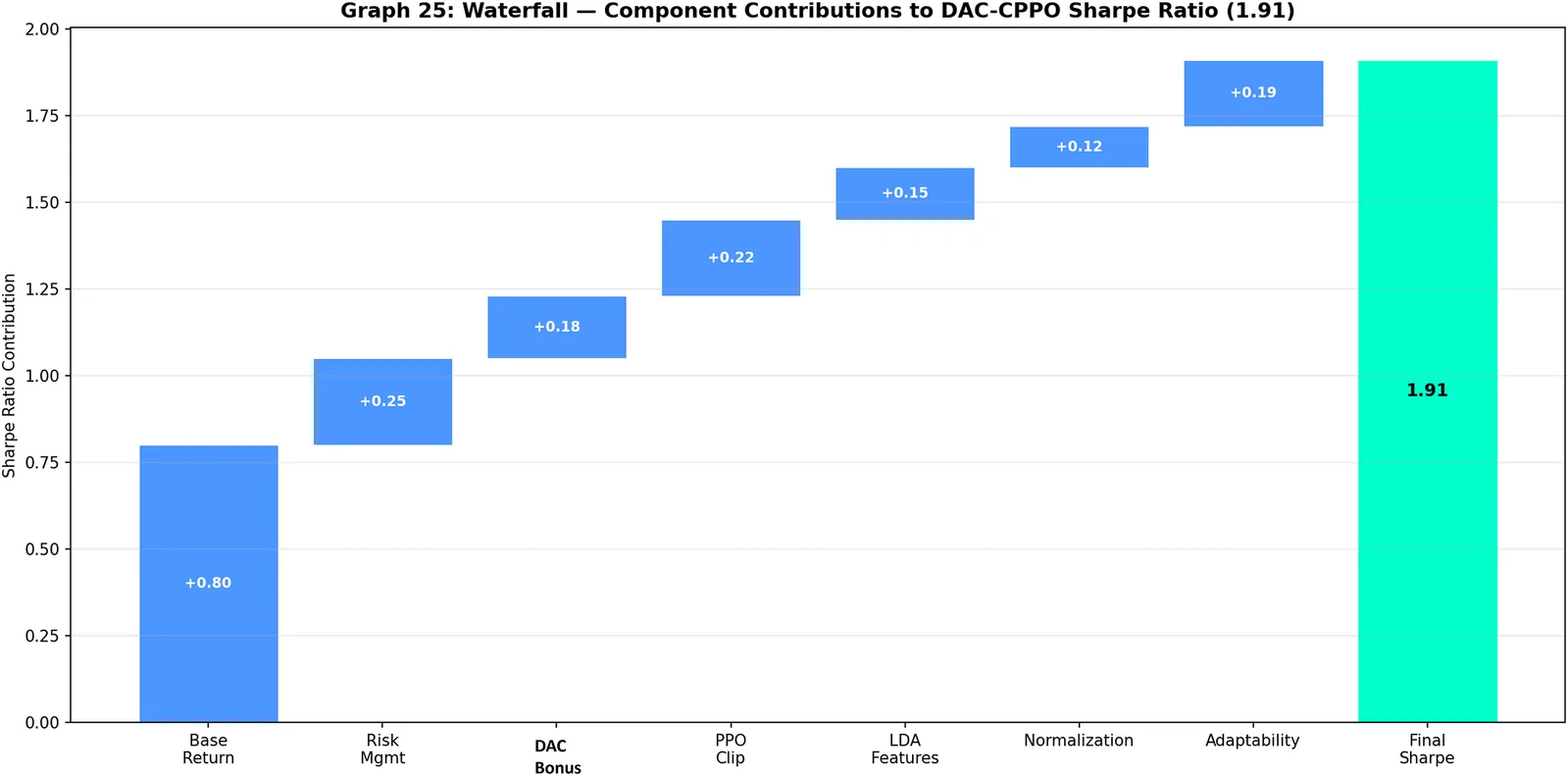
Component contributions to DAC-CPPO sharpe ratio for financial optimization.

Fig 10 (a) visualizes the green candles to show an increase in price (closing price is higher than the opening price), and red candles to show a decrease in price (closing price is lower than the opening price). This is the price trend of every trading period. The volume subplot below the candlestick chart in Fig 10 (b) displays the volume of each session, and the bars in the plot are green and red, meaning higher buying volume and selling volume, respectively, which gives one an idea of the activity in the market in each trading period.

**Fig 10:**
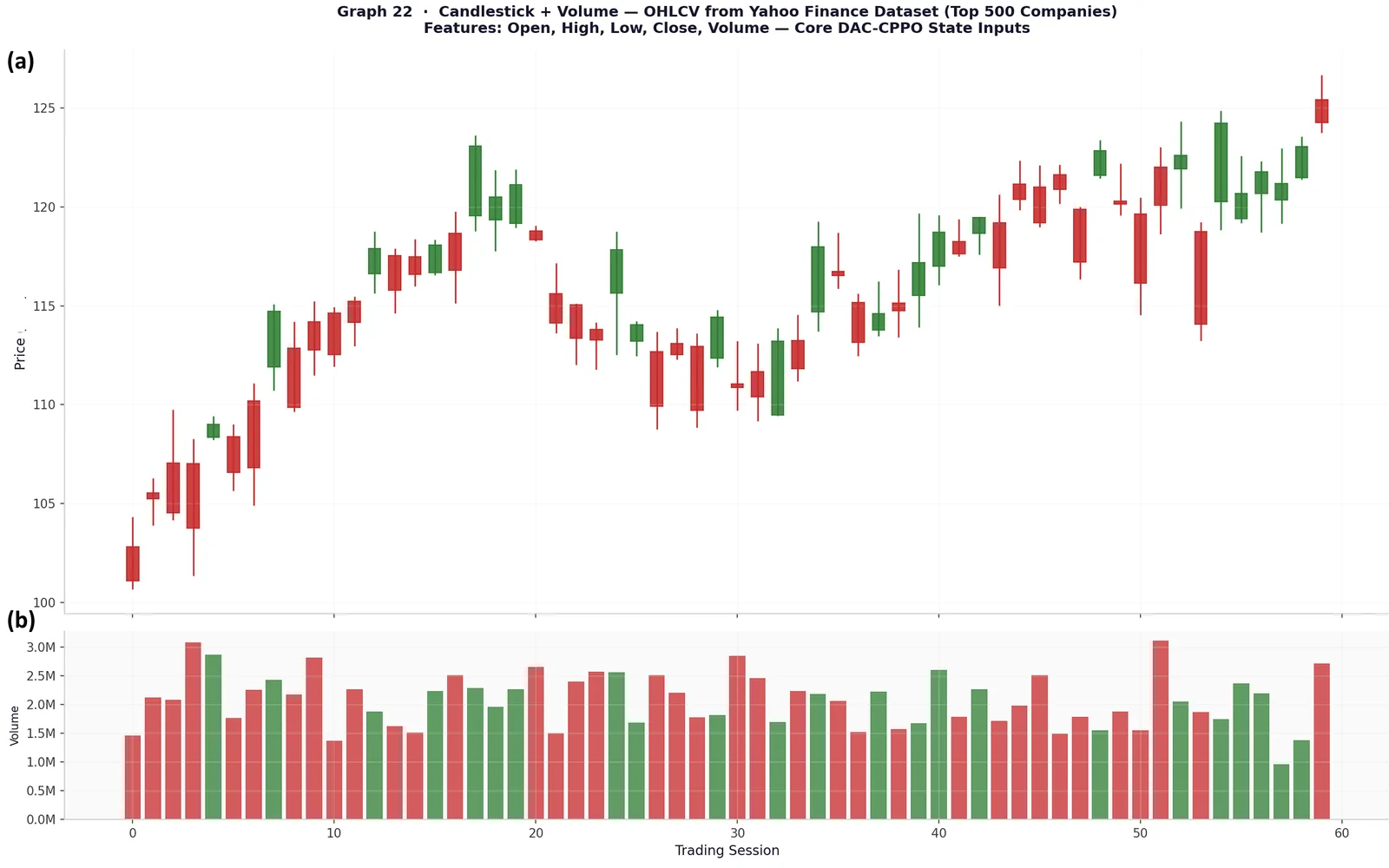
Data features of stock prices for top 500 companies (a) stock price movements (b) trading activity levels.

The Pearson correlation between different financial features and three types of Capital Action (Increase, Hold, and Decrease) is shown in this heatmap. Fig 11 shows the visualization of the heatmap. There are strong positive relationships with the LDA1, MACD, and Sharpe Ratio with the Increase class being observed, and they are thus important predictors. Contrarily, several features are negatively linked to “Decrease,” such as “Sentiment” and a “Sharpe_Ratio,” and reveal the inverse relations within the market trends.

**Fig 11:**
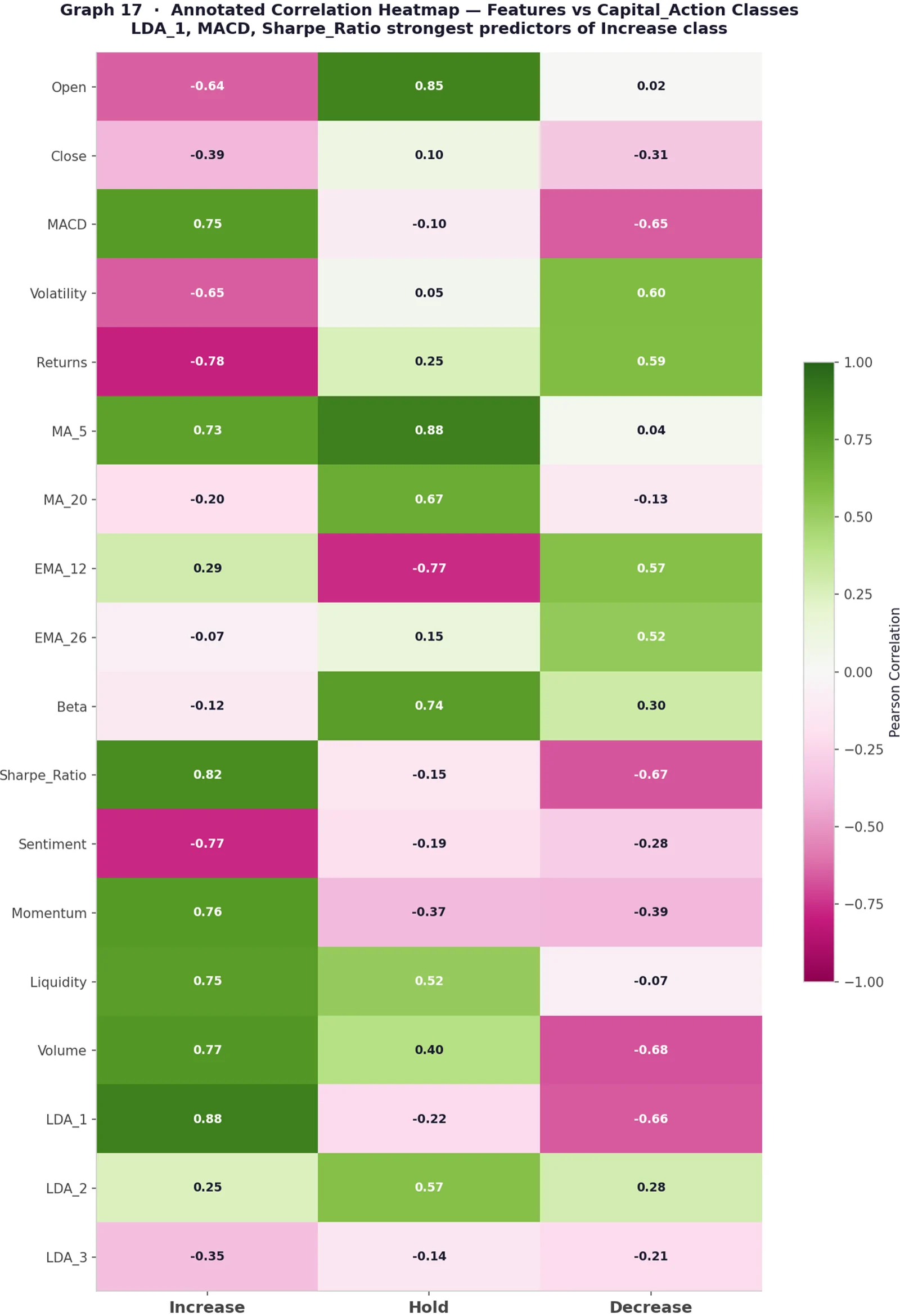
Features correlation heatmap with capital action classes in stock analysis.

The arrangement of main characteristics of the stock market of the top 500 companies, mostly concentrating on data attributes, including Open, Close, High, Low, Volume, Dividends, and Stock Splits, with separate piecewise models of such companies as AAPL, MSFT, GOOGL, AMZN, and NVDA. This is depicted in Fig 12. The distribution of each feature is graphically obtained with time, and particularly how the price and trading conduct fluctuate amongst various companies. The analysis assists in evaluating the trends within the market and performance within the company.

**Fig 12:**
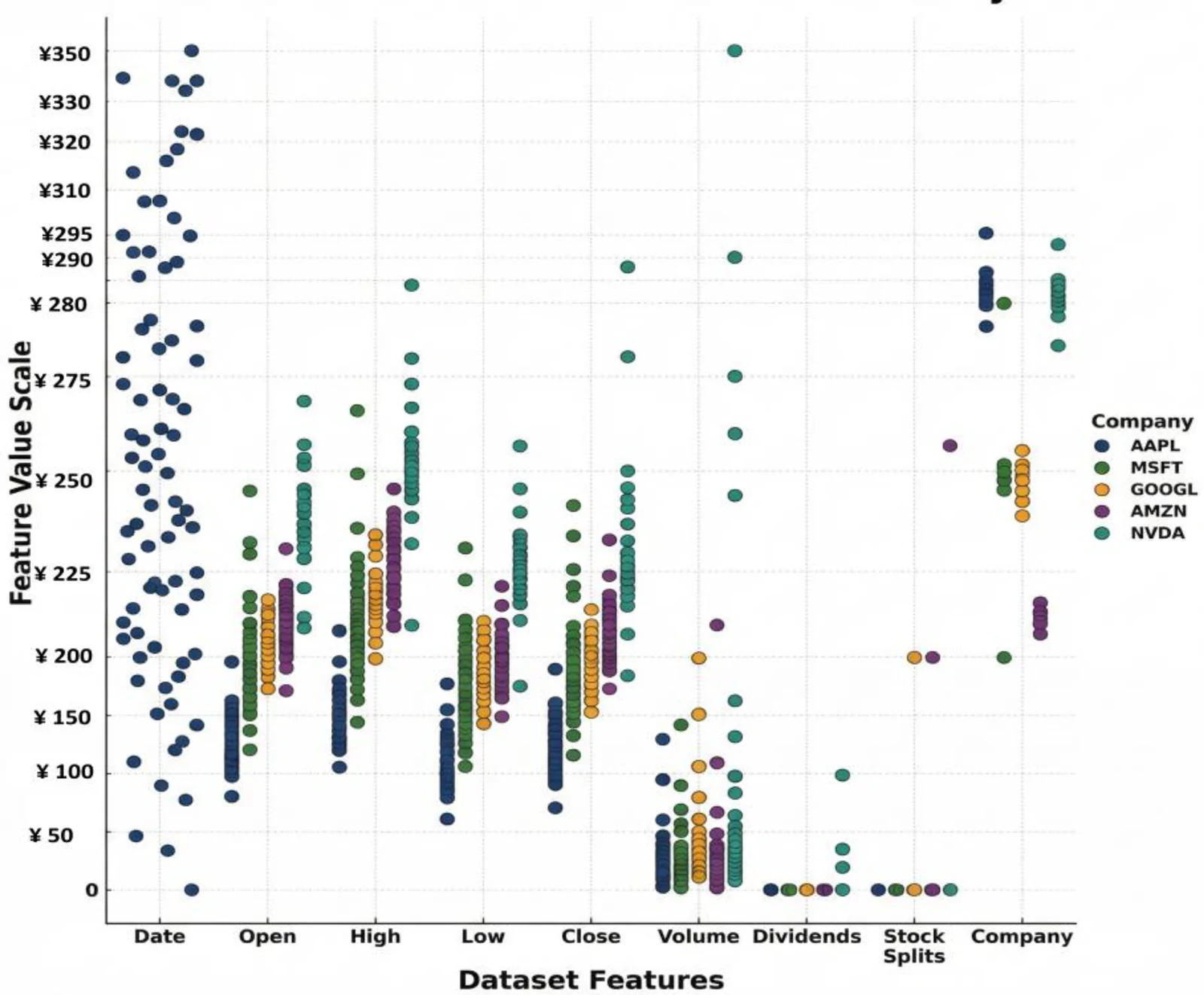
Stock market feature distribution for top 500 companies (price, volume).

### 3.2. Comparative analysis

Table 3 provides the key measures and their formulas used to evaluate the performance of predictive models in this research. In this research, these metrics are applied to assess both prediction accuracy and the risk-aware decision quality of the proposed DAC-CPPO reinforcement learning model for optimal capital allocation in dynamic financial markets. Accuracy captures how accurate the market action sets are, and how well the model does at capturing the optimal action of an investment. Precision filters out false signals for profit while Recall captures the ability to identify all profitable signals. F1-Score balances the two for responsible decision making, while RMSE and MSE penalize for large errors in the predicted results, stabilizing performance in distressed market conditions. MAE measures the prediction average deviation for broad forecasting accuracy while MAPE measures based on percentage error comparisons between assets. R-Squared measures how well the model outperforms naive market models based on standard deviation of the financial return deviations.

**Table 3:** Key evaluation metrics and formulas for model performance assessment.

Metrics	Formula	Description
**Accuracy**	$\frac{T_{P}+T_{N}}{T_{P}+T_{N}+F_{P}+F_{N}}$ T,F−TrueandFalsevalues PandN−PositiveandNegativevalues	Proportion of correct predictions; higher values indicate better overall model performance.
**Precision**	$\frac{T_{P}}{T_{P}+F_{P}}$	Measures correct positive predictions; higher values indicate fewer false positives in predictions.
**Recall**	$\frac{T_{P}}{T_{P}+F_{N}}$	Measures ability to correctly identify all actual positive instances.
**F1-Score**	$\text{2}\times \frac{Precison\times Recall}{Precison+Recall}$	Balances precision and recall to evaluate overall positive prediction performance.
**RMSE**	$\sqrt{\frac{\text{1}}{n}\sum_{i=\text{1}}^{n}(y_{i}-\hat{y_{i})}}$ $y_{i}-$actual value, ${\hat{y}}_{i}$predicted value, $\overset{―}{y}$mean of actual values,i−data point index and n – number of observations	Measures square root of average squared prediction errors, emphasizing larger financial forecasting deviations.
**MAPE**	$\frac{\text{100}}{n}\sum_{i=\text{1}}^{n}\left|\frac{(y_{i}-\hat{y_{i})}}{y_{i}}\right|$	Evaluates percentage-based prediction error, useful for comparing forecasting accuracy across financial assets.
**MAE**	$\frac{\text{1}}{n}\sum_{i=\text{1}}^{n}|y_{i}-{\hat{y}}_{i}|$	Computes average absolute difference between predicted and actual values, indicating prediction accuracy.
**R-Square**	$\text{1}-\frac{\sum_{i=\text{1}}^{n}{(y_{i}-\hat{y_{i})}}^{\text{2}}}{\sum_{i=\text{1}}^{n}{(y_{i}-\overset{―}{\overset{―}{y})}}^{\text{2}}}$	Indicates proportion of variance explained by model, reflecting predictive capability and reliability.
**MSE**	$\frac{\text{1}}{n}\sum_{i=\text{1}}^{n}({y_{i}-{\hat{y}}_{i})}^{\text{2}}$	Measures mean squared differences between predicted and actual values, penalizing large errors.

The DAC-CPPO algorithm is compared against two groups of reference algorithms. The first group consists of conventional ML and DL models (LR, RF, GB, SVM, LSTM, and Transformer), which are all directly applicable to financial forecasting and portfolio selection problems. The other group of algorithms is a set of financial sentiment models, i.e., FinBERT, BERT-base-uncased, RoBERTa-base, VADER, and TextBlob. The aforementioned sentiment models are used as indirect reference algorithms since they are extensively applied in intelligent investment frameworks for generating market sentiment that would later guide trading decisions. As the above-mentioned sentiment models were initially created to deal with financial text sentiment classification, not portfolio allocation/capital actions prediction per se, their performance can only serve as an indirect reference. Table 4 depicts the comparative performance of financial prediction techniques and sentiment analysis techniques along with the proposed DAC-CPPO model. Traditional machine learning techniques RF [30], LSTM [30], SVM [30], and GB [30] and deep learning models (Long Short-Term Memory and Transformer) act as baseline references for the financial prediction task, whereas, FinBERT, BERT-base-uncased, RoBERTa-base, VADER, and TextBlob act as financial sentiment analysis techniques which are frequently used with intelligent investment systems to help understand the market. These sentiment analysis techniques can be considered indirect reference baselines due to the fact that they do not directly allocate capital like the proposed DAC-CPPO model. The proposed DAC-CPPO model outperforms all other baseline techniques with the best accuracy (97.6%), precision (97.1%), recall (96.5%), F1-score (96.9%), and AUC-ROC (0.984).

**Table 4:** Comparative performance of ML approaches and the proposed DAC-CPPO model.

Model	Dataset	Accuracy (%)	Precision (%)	Recall (%)	F1-Score (%)	AUC-ROC
FinBERT [29]	FNSPID Financial News Data Set	88	87	87	88	–
BERT-base-uncased [29]		81	80	80	81	–
RoBERTa-base [29]		83	82	82	83	–
VADER [29]		65	63	64	66	–
TextBlob [29]		63	62	61	63	–
LR [30]	Bloomberg, Yahoo Finance, Quandl, and Kaggle	82.4	81.5	79.8	80.6	0.85
RF [30]		88.7	87.9	86.3	87.1	0.91
GB [30]		91.2	90.4	89.6	90.0	0.94
SVM [30]		85.6	84.8	83.2	84.0	0.88
LSTM [30]		93.4	92.7	91.5	92.1	0.96
Transformer [30]		94.8	94.1	93.0	93.5	0.97
DAC-CPPO [Proposed]	Intelligent Finance Assets and Massive Yahoo Finance dataset (Kaggle)	97.6	97.1	96.5	96.9	0.984

Table 4 offers the comparison results of several sentiment analysis and machine learning schemes to perform financial prediction tasks. Conventional algorithms like VADER [29] and TextBlob [29] are not as efficient since they do not have much understanding of their context. Moderate improvements are made by classical ML models such as LR [30], RF [30], GB [30], SVM [30], etc. DL models such as LSTM [30] and Transformer architecture substantially improve prediction accuracy and classification reliability. The proposed DAC-CPPO model achieves the highest performance across all metrics, demonstrating superior accuracy (97.6%), precision (97.1%), recall (96.5%), F1-score (96.9%), and an AUC-ROC of 0.984 for intelligent financial sentiment. The comparison between the suggested DAC-CPPO algorithm and classical machine learning or deep learning methods which directly aim at financial forecasting is presented in Fig 13. The comparative illustration for the case of indirect comparison with models which are used for financial sentiment analysis and are applied in the intelligent investment pipeline is depicted in Fig 14.

**Fig 13:**
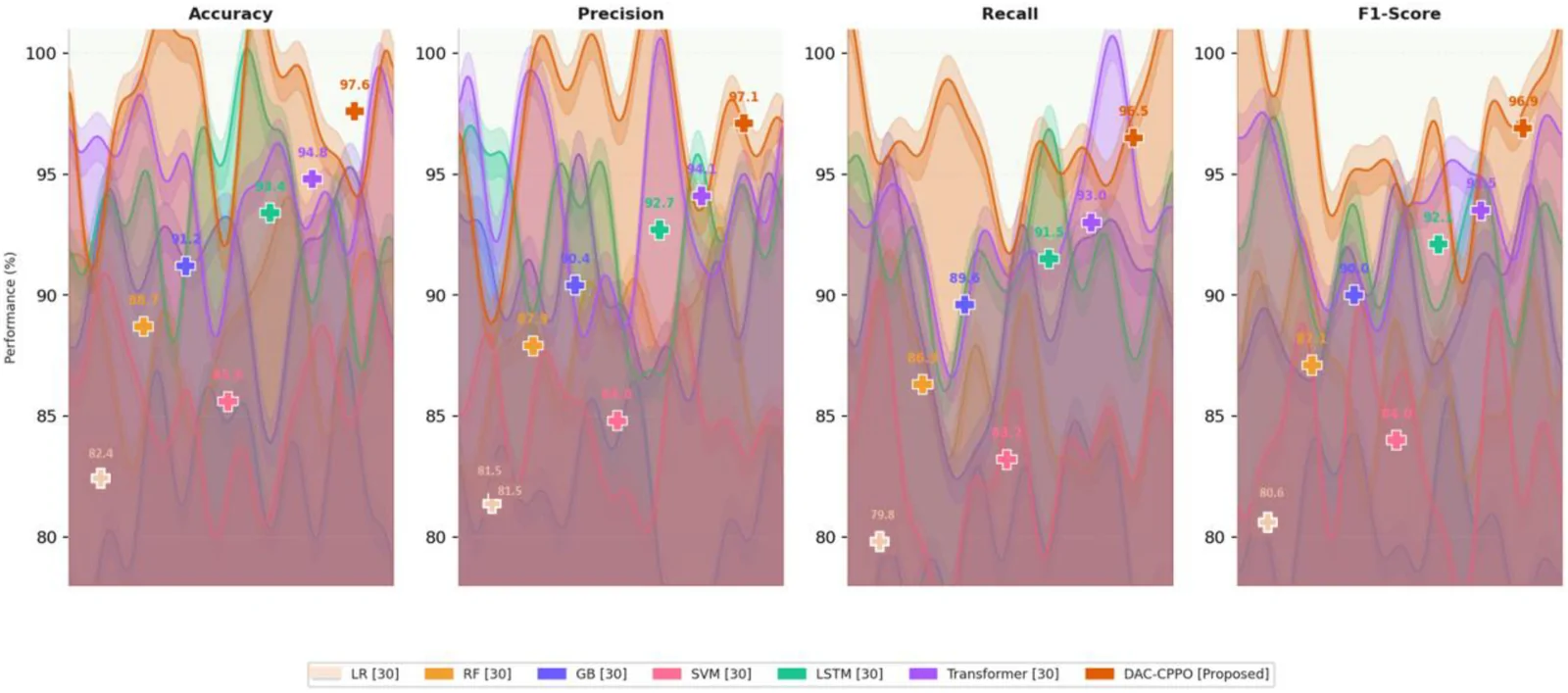
Performance comparison of existing models [30] and proposed DAC-CPPO visualization results.

**Fig 14:**
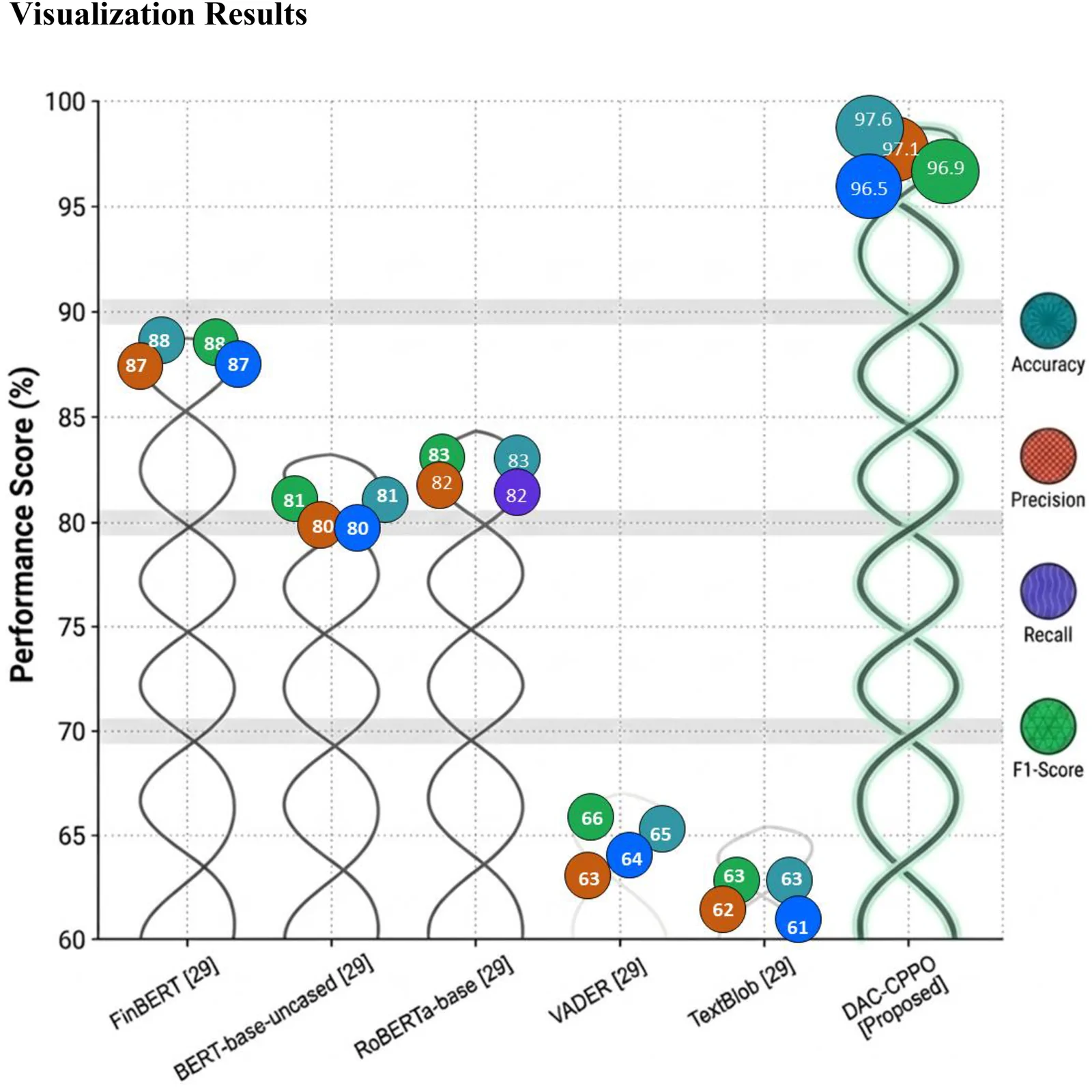
Visualization performance comparison of existing models [29] and proposed DAC-CPPO model.

Fig 14 shows the performance comparison of the selected financial sentiment analysis methods that include FinBERT, BERT-base-uncased, RoBERTa-base [29], VADER [29], and TextBlob [29] along with the proposed DAC-CPPO method. The models mainly do financial sentiment classification and thus function as indirect reference baseline algorithms rather than direct portfolio allocation strategies. Their representation serves to show the potential of financial sentiment-based intelligence methods compared to other intelligent investing systems while DAC-CPPO is focused on capital allocation through reinforcement learning. The relative performance of FinBERT, BERT-base-uncased, RoBERTa-base, VADER, TextBlob, and the suggested DAC-CPPO model is depicted in Fig 14, which shows that the suggested model gives the best performance among all the metrics used. Table 5 provides the Comparative evaluation of error metrics, showing improved prediction accuracy and reliability of the proposed DAC-CPPO model against traditional, machine learning, and deep learning approaches.

**Table 5:** Comparison of error metrics across various models with the proposed DAC-CPPO model.

Model	MAE	MSE	RMSE	MAPE	R-squared
Traditional Model [28]	–	–	0.182	11.4	–
SVM [28]	–	–	0.114	6.9	–
LR [30]	1.72	0.045	0.067	–	0.84
RF [30]	1.28	0.031	0.056	–	0.91
GB [30]	1.12	0.027	0.052	–	0.93
LSTM [30]	0.98	0.021	0.046	–	0.95
Transformer [30]	0.91	0.018	0.042	–	0.97
DAC-CPPO [Proposed]	0.074	0.013	0.081	4.6	0.985

Note: The error values provided for the baseline models in Table 5 were extracted from their corresponding references ([28,30]). As the experiments used distinct datasets and preprocessing techniques, it is worth noting that the values of MAE, MSE, RMSE, MAPE, and R² have been reported in their original units of measurement and thus cannot be compared with one another. The new DAC-CPPO framework was tested on the Intelligent Finance Assets and Massive Yahoo Finance datasets according to the experimental setup.

Table 5 and Fig 15 indicate the performance measure of different techniques, such as Traditional models [28], tree-based models like Random Forest and Decision Tree, which are conventional machine learning algorithms used as baseline methods for performance comparison, RF [30], SVM [30], LSTM [30], and Transformer. The DAC-CPPO model is the best in terms of the lowest MAE (0.074), MSE (0.013), and the RMSE (0.081), which means that the prediction accuracy is high. The model also has good performance, as indicated in its MAPE (4.6) and R-squared (0.985); it has the potential to maximize risk-adjusted returns and minimize the level of mistakes made in forecasting the portfolio in relation to other models. All experiments are conducted using a 5-year historical financial dataset covering market records from the Massive Yahoo Finance dataset, ensuring long-term market dynamics are captured for robust evaluation.

**Fig 15:**
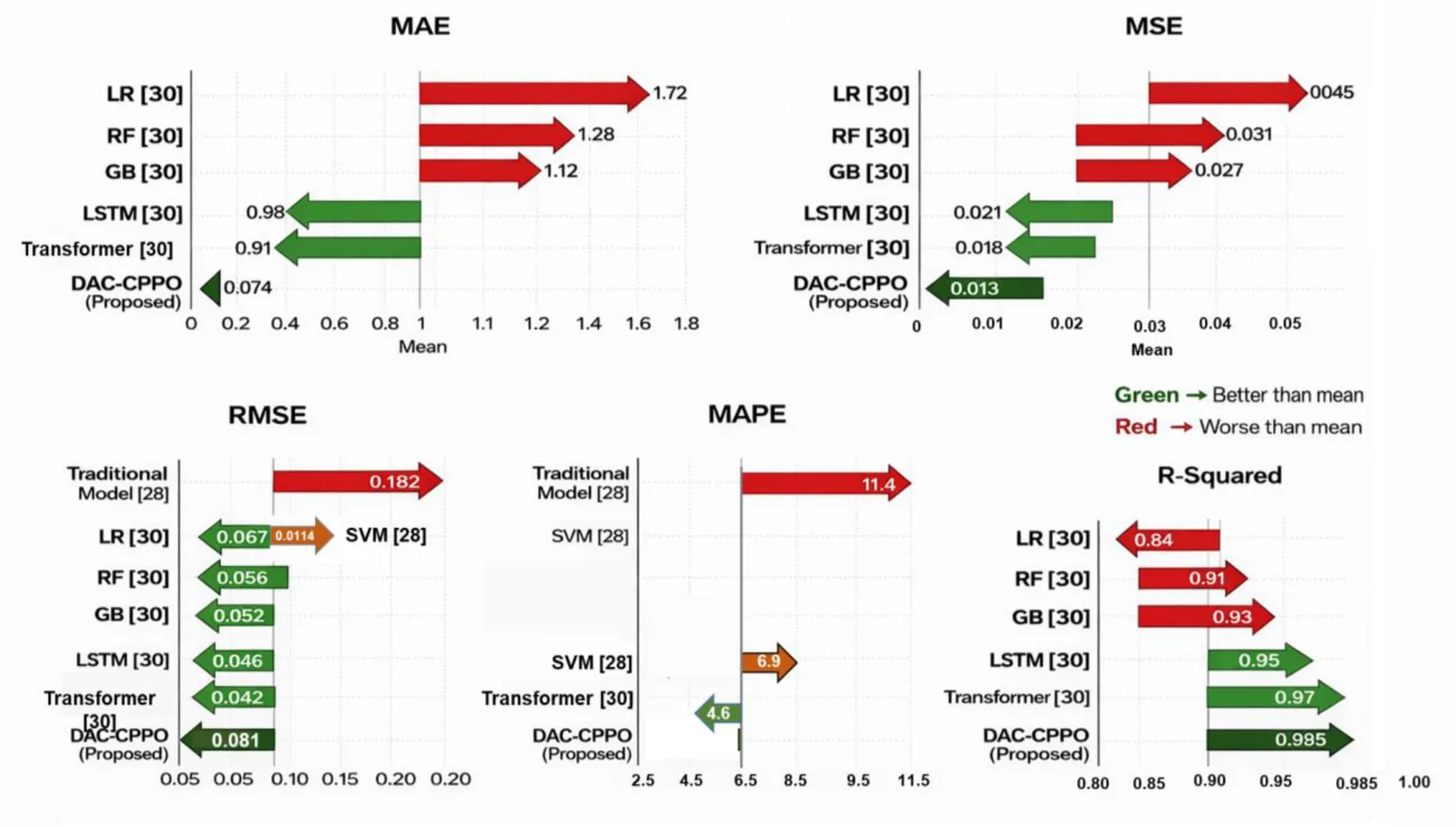
Visualization of prediction error metrics comparing existing models and proposed DAC-CPPO framework performance.

The comparative transaction of Sharpe ratios under different models is listed in Table 6 and Fig 16, indicating the excellence of the suggested DAC-CPPO. Sharpe ratio is a ratio that is used in measuring portfolio risk-adjusted returns, which measures excess returns per unit increase in volatility, whereby higher values imply excellent performance, enhanced efficiency, and also enhanced investment stability. The Sharpe ratio is computed using the following equation (19).

**Table 6:** Comparison of sharpe ratios for various models and proposed DAC-CPPO framework.

Model	Sharp Ratio
**LR** [30]	1.21
**RF** [30]	1.36
**GB** [30]	1.48
**LSTM** [30]	1.59
**Transformer** [30]	1.72
**Traditional Model** [28]	0.88
**SVM** [28]	1.19
**DAC-CPPO [Proposed]**	1.91

**Fig 16:**
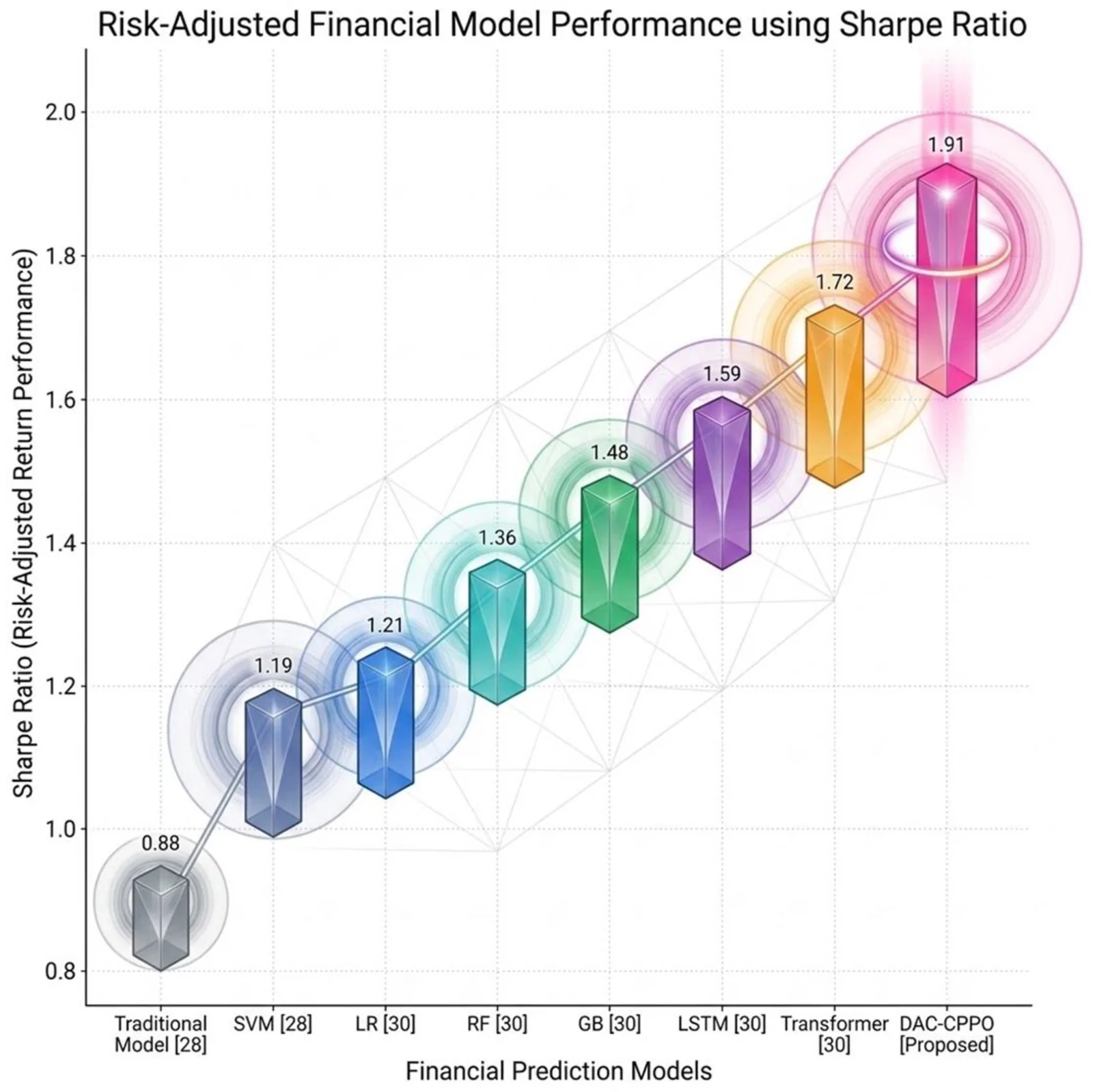
Comparative sharpe ratio performance of financial models and proposed DAC-CPPO framework.


$$\begin{array}{c} S=\frac{R_{p}-R_{f}}{\sigma_{p}} \end{array}$$ (19)

where SRdenotes the annualized Sharpe ratio, $R_{p}$is the average daily portfolio return, $R_{f}$is the annual risk-free rate, and $\sigma_{p}$is the standard deviation of daily portfolio returns. The Sharpe ratio was computed using daily portfolio returns and annualized using 252 trading days according to $SR=\sqrt{\text{2}\text{5}\text{2}}\times \frac{\bar{R_{p}-R_{f}}}{\sigma_{p}}$. An annual risk-free rate of 2% (0.02) was adopted throughout all experiments. The same Sharpe ratio calculation procedure was consistently applied to the proposed DAC-CPPO model and all baseline methods to ensure fair comparison across Table 6 and Fig 16.

All Sharpe ratios used here are annual numbers, calculated using the daily returns of the portfolio on 252 trading days per year with an annual risk-free rate of 2% (0.02).

These reported Sharpe ratios are annualized ratios that have been calculated from daily returns for each portfolio with 252 trading days and an annual risk-free rate of 2% (0.02). The performance comparison of various traditional ML, DL, and DRL models for intelligent capital allocation is presented in Table 7. All models, including DQN [27], Traditional methods, SVM [28], FinBERT, BERT-base-uncased, VADER [29], LR, RF, GB, LSTM, Transformer [30], and the proposed DAC-CPPO method, were implemented and trained using two benchmark datasets: the Intelligent Finance Assets dataset and the Massive Yahoo Finance dataset, ensuring a fair and consistent evaluation environment across all approaches. The results demonstrate that traditional sentiment and statistical models, such as VADER and Logistic Regression, achieve comparatively lower performance due to their limited capability in capturing complex nonlinear financial dependencies. Machine learning models such as SVM, RF, and GB show improved predictive ability, while deep learning architectures, including LSTM and Transformer models, further enhance performance by effectively modeling sequential financial patterns. The DRL-based DQN model demonstrates better decision-making capability compared to classical ML and DL models, highlighting the advantage of reinforcement learning in dynamic financial environments.

**Table 7:** Comparative performance of ML and DL techniques on financial datasets for capital allocation.

Models	Accuracy (%)	Precision (%)	Recall (%)	F1-Score (%)
**DQN**	89.12	88.65	89.84	87.36
**Traditional Model**	92.11	91.13	90.89	92.50
**SVM**	90.10	91.00	90.80	91.10
**FinBERT**	93.00	83.00	83.20	84.00
**BERT-base-uncased**	67.00	66.00	66.80	68.50
**VADER**	84.50	83.70	81.80	82.60
**LR**	90.20	89.40	89.10	89.70
**RF**	92.30	91.30	90.50	91.30
**GB**	87.70	86.90	85.50	86.20
**LSTM**	95.10	94.40	93.90	95.20
**Transformer**	94.20	93.50	92.80	93.60
**DAC-CPPO [Proposed]**	97.60	97.10	96.50	96.90

Table 7 provides that the proposed DAC-CPPO model significantly outperforms all baseline approaches by achieving the highest Accuracy (97.6%), Precision (97.1%), Recall (96.5%), and F1-Score (96.9%). This improvement enhances policy stability, exploration efficiency, and risk-sensitive decision-making. Overall, the results confirm that the proposed DAC-CPPO method provides superior adaptability, robustness, and risk-adjusted performance for intelligent capital allocation in volatile financial markets.

### 3.3. Class-wise performance analysis

Despite the proposed DAC-CPPO model having an overall accuracy of 97.6%, it is still necessary to conduct a further class-level test to confirm that the model is equally effective for all the portfolio decision-making classes. The test was done using the chronological testing set in which the portfolio decisions were classified as Increase, Hold, and Decrease. Precision, recall, and F1-score of each class were considered together with the confusion matrix.

As shown in Fig 17, the classification is quite good for all three portfolio actions. In case of actual “Increase” actions, there are 4 correct classifications while 1 is misclassified as “Hold”. In case of actual “Hold” actions, there are 3 correct classifications, with 1 being misclassified as “Increase” action and another 1 as “Decrease” action.

**Fig 17:**
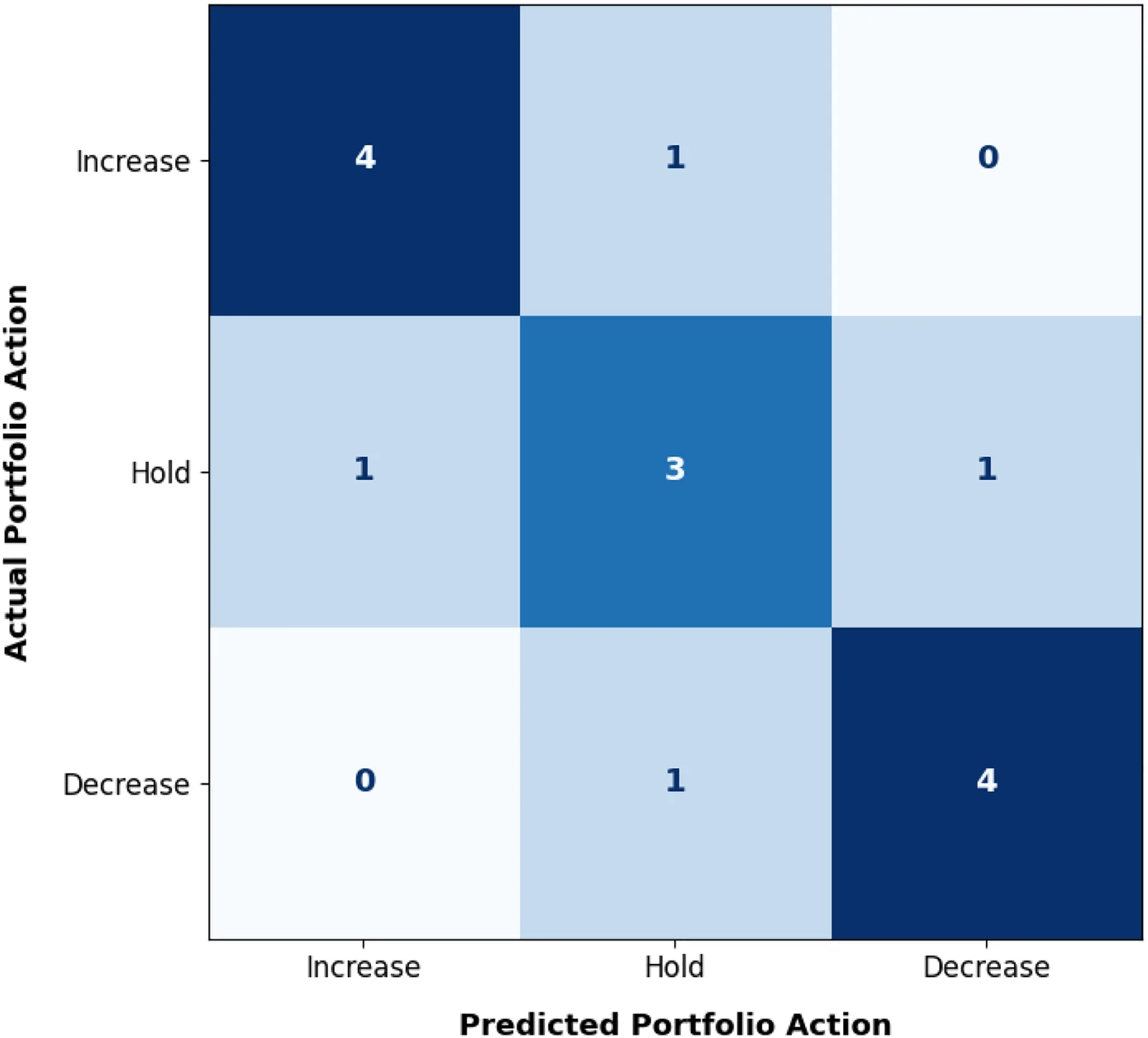
Confusion matrix of the proposed DAC-CPPO model for portfolio action classification.

### 3.4. Ablation research

The ablation research assesses the impact of every building block of architecture on the experimental results. Preprocessing, feature extraction, DAC learning, and CPPO stabilization are presented results respectively by turn. Results indicate that reinforcement learning parts has contributed to the security of financial decision stability, the ability of risk control, and the efficiency of rate of return. Ablation research results signify that the architecture of DAC-CPPO configuration can balance the risk adjusted return performance and the prediction accuracy better. The contribution of preprocessing, feature extraction, DAC and CPPO is illustrated in Table 8.

**Table 8:** Ablation analysis of proposed DAC-CPPO framework for intelligent portfolio allocation.

Model Variant	CPPO	Pre-processing	Feature Extraction	DAC	Cumulative Return	Volatility	Sharpe Ratio	Accuracy (%)
**Baseline CPPO**	✓				0.42	0.31	0.91	78.5
**+ Pre-processing**	✓	✓			0.55	0.27	1.10	84.6
**+ Feature Extraction**	✓	✓	✓		0.71	0.23	1.30	90.2
**DAC-CPPO (Proposed)**	✓	✓	✓	✓	1.12	0.14	1.91	97.6

Table 8 presents an ablation analysis evaluating the contribution of each component in the proposed framework. Starting with only preprocessing, performance gradually improves as feature extraction, DAC learning, and CPPO stabilization are added. Each additional module increases cumulative return and Sharpe ratio while reducing volatility. The complete DAC-CPPO model achieves the best results with 1.12 cumulative return, 1.91 Sharpe ratio, and 97.6% accuracy.

### 3.5. Time-series cross-validation analysis

To perform a statistically sound validation that takes into account the chronological nature of the financial market data set, the DAC-CPPO framework was validated using an approach of 5-fold expanding window time-series cross-validation. Unlike traditional random k-fold cross-validation, which introduces the problem of look-ahead bias through the inclusion of future observations in the training set, the used validation method preserves the temporal ordering for all the folds. In each round of the algorithm, the model was trained with all the historical observations and tested on the following unseen time segment. After the completion of each fold, the training set was extended with the previous testing observation before performing the test for the next unseen time interval. This validation method represents the actual approach of portfolio management, in which investment is performed only on the basis of the historical data. The average performance of the proposed model on the validation folds was reported using mean, SD, 95% CI and paired t-test is represented as Table 9.

**Table 9:** Cross-validation performance evaluation of DAC-CPPO model for reinforcement learning-driven optimal capital allocation in intelligent finance.

Metric	Mean (%)	SD	95% CI	p-value	Significance
**Accuracy**	97.4	0.62	[96.78 – 98.02]	<0.001	Significant
**Precision**	97.0	0.68	[96.32 – 97.68]		
**Recall**	96.2	0.74	[95.46 – 96.94]		
**F1-Score**	96.5	0.70	[95.80 – 97.20]		

The performance results for the suggested DAC-CPPO model through the execution of the 5-fold expanding window time series cross-validation method are provided in Table 9. The model managed to score a mean accuracy of 97.4%, a precision of 97.0%, a recall of 96.2%, and an F1-score of 96.5%. This shows that the proposed model has reliable prediction capabilities regardless of the chronological partitioning of the financial market. The small standard deviation and the tight 95% confidence intervals show that the generalization ability of the proposed model does not change with each consecutive partitioning of the financial market data. In addition, the p-value from the paired t-test is less than 0.001, which demonstrates statistical significance in the improvement.

### 3.6. Discussion

The discussion section evaluating the proposed DAC-CPPO model with better portfolio allocation performance, higher risk-adjusted return, lower volatility and more reliable decision stability is to make far more explicit. limitations of most existing portfolio allocation models. Big data finance models increase the utilization of information; however, they are highly susceptible to the quality and infrastructure of data (lowering the resilience in practice). The model design proposed implements the preprocessing (data normalization, data cleaning, LDA) to produce robust inputs. Carbide clustering models are also optimized by IoT schemes to enhance segmentation, but do not directly optimize risk-adjusted optimization, which constrains financial learning effectiveness; DAC-CPPO is a direct maximization of long-term Sharpe goals, risk-sensitive capital allocation. Traditional DRA methods can be weak in terms of risk adjustment, with respect to using non-stationary markets, and residential to unstable allocations are acquired in situations where tail risk is present [28]; the suggested DAC-CPPO framework combines risk-sensitive rewards and clipped updates to bring about a high-quality decision. Sentiment enhancement DRL models are expressive, but have high computational complexity and low multi-asset generalization [29]. The model algorithm proposed here trades expressivity and scalable policy learning. Third, traditional machine learning systems can provide predictive accuracy but no dynamic feedback control, which undermines adaptive allocation [30]; DAC-CPPO was an actor-critic system, where the weights are always updated. The proposed model of DAC-CPPO has feasible benefits in introducing smart capital allocation to the changing financial market. It assists in balancing the exploration of the DAC as well as that of maintenance of the policy, which comprises the adaptive rebalancing of the portfolio, risk-adjusted returns, and exposure to volatility. Financial institutions and asset managers can use this approach to enhance automated investment processes, capital allocation, and provide information-based finance decisions.

The high accuracy rate obtained using the DAC-CPPO framework needs to be understood in conjunction with the evaluation methodology used. The problem of financial time series forecasting is intrinsically difficult due to factors such as market volatility, temporal dependencies, and non-stationarity. To have a proper evaluation, train-test splits were performed chronologically and no feature extraction used observations beyond those available at each point prior to forecasting. Also, class-wise analysis reveals reliable forecasting capabilities for the Increase, Hold, and Decrease portfolio decisions, and not just one dominant class. Such evaluations are evidence that the results obtained indicate good learning abilities of the proposed framework for portfolio decision-making in dynamic financial markets and not just luck of data leakage or an easy classification problem.

## 4. Conclusion

Optimal capital allocation in dynamic financial markets was examined using reinforcement learning. Intelligent Finance Assets and a Massive Yahoo Finance dataset were collected from publicly available financial sources. The data were preprocessed, and key financial features were extracted using LDA to enhance model performance. The research implemented the DAC-CPPO model for portfolio optimization, achieving 97.6% accuracy, 0.074 MAE, 0.081 RMSE, and a 1.91 Sharpe ratio, 1.12 Cumulative returns, and 0.14 Volatility. These results demonstrate the model's robust risk-adjusted performance associated with traditional approaches. The proposed DAC-CPPO model effectively addressed the challenges of dynamic market environments, offering higher returns and robust risk management, proving its potential for practical capital allocation.

The DAC-CPPO model's limitations include computational complexity, dependency on high-quality data, and challenges in real-time deployment for high-frequency trading. Further research should focus on enhancing scalability, improving robustness during extreme market events, and addressing model interpretability to ensure practical application in dynamic financial environments. Exploring multi-asset strategies and real-time feedback integration will further refine the model’s capabilities.

## Supporting information

S1 File Appendix table. (DOCX)

S2 File Code. (ZIP)
