# Supplementary material for: Reinforcement learning-driven optimal capital allocation for intelligent finance
Source: PLoS One. 2026 Oct 20;21(10):e0348010. doi: 10.1371/journal.pone.0348010 (PMC13682667; doi:10.1371/journal.pone.0348010)
Supplement: Supplementary file 1 [file pone.0348010.s001.docx]

**APPENDIX TABLE**

| $\boldsymbol{ACCRONYM}$ | $\boldsymbol{FULL FORM}$ |
| --- | --- |
| $GB$ | $Gradient Boosting$ |
| $SVM$ | $Support Vector Machine$ |
| $LR$ | $Logistic Regression$ |
| $RF$ | $Random Forest$ |
| $Financial BERT$ | $Financial Bidirectional Encoder Representations from Transformers.$ |
| $BERT-base$ | $Bidirectional Encoder Representations from Transformers (base version)$ |
| $RoBERTa$ | $Robustly optimized Bidirectional Encoder Representations from Transformers. approach$ |
| $VADER$ | $Valence Aware Dictionary and Sentiment Reasoner$ |
| $AI$ | $Artificial Intelligence$ |
| $ML$ | $Machine Learning$ |
| $DRL$ | $Deep Reinforcement Learning$ |
| $LSTM$ | $Long Short-Term Memory$ |
| $ANN$ | $Artificial Neural Network$ |
| $NLP$ | $Natural Language Processing$ |
| $DQN$ | $Deep Q-Network$ |
| $DDPG$ | $Deep Deterministic Policy Gradient$ |
| $CVaR$ | $Conditional Value at Risk$ |
| $DCF$ | $Discounted Cash Flow$ |
| $ROI$ | $Return on Investment$ |
| $IoT$ | $Internet of Things$ |
| $ANOVA$ | $Analysis of Variance$ |
| $MAE$ | $Mean Absolute Error$ |
| $MSE$ | $Mean Squared Error$ |
| $RMSE$ | $Root Mean Squared Error$ |
| $MAPE$ | $Mean Absolute Percentage Error$ |
| $R-squared$ | $Coefficient of Determination$ |
| $MDP$ | $Markov Decision Process$ |
| $TD error$ | $Temporal Difference error.$ |
| $DAC$ | $Asynchronous advantage Actor-Critic$ |
| $RL$ | $Reinforcement learning$ |
| $PPO$ | $Proximal Policy Optimization$ |
| $LDA$ | $Linear Discriminant Analysis$ |
| $MACD$ | $Moving Average Convergence Divergence$ |
| $AUC$ | $Area Under the Curve$ |
| $ROC$ | $Receiver Operating Characteristic$ |
| $CHMC$ | $Cognitive Helix Metric Constellation$ |
| $LLM$ | $Large Language Model$ |
